# Application of fused-grid-based CYP-Template systems for genotoxic substances to understand the metabolisms

**DOI:** 10.1186/s41021-023-00275-4

**Published:** 2023-08-07

**Authors:** Yasushi Yamazoe, Norie Murayama, Tomoko Kawamura, Takashi Yamada

**Affiliations:** 1 Division of Drug Metabolism and Molecular Toxicology, Graduate School of Pharmaceutical Sciences, Tohoku University, 6-3 Aramaki-Aoba, Aoba-ku, Sendai, 980-8578 Japan; 2 Division of Risk Assessment, Center for Biological Safety and Research, National Institute of Health Sciences, 3-25-26 Tonomachi, Kawasaki-ku, Kawasaki, 210-9501 Japan; 3 Showa Pharmaceutical University, Machida, Tokyo, 194-8543 Japan

**Keywords:** CYP-mediated activation, P450 induction, Distinction between human and rodent CYP1A1, Regioselectivity and placement on Template, Enhancement and simultaneous bi-molecule sittings, Co-mutagenicity, Fused hexagonal-grid Template system

## Abstract

Understanding of metabolic processes is a key factor to evaluate biological effects of carcinogen and mutagens. Applicability of fused-grid Template* systems of CYP enzymes (Drug Metab Pharmacokinet 2019, 2020, 2021, and 2022) was tested for three phenomena. (1) Possible causal relationships between CYP-mediated metabolisms of β-naphthoflavone and 3-methylcholanthrene and the high inducibility of CYP enzymes were examined. Selective involvement of non-constitutive CYP1A1, but not constitutive CYP1A2, was suggested on the oxidative metabolisms of efficient inducers, β-naphthoflavone and 3-methylcholanthrene. These results supported the view of the causal link of their high inducibility with their inefficient metabolisms due to the lack of CYP1A1 in livers at early periods after the administration of both inducers. (2) Clear differences exist between human and rodent CYP1A1 enzymes on their catalyses with heterocyclic amines, dioxins and polyaromatic hydrocarbons (PAHs). Reciprocal comparison of simulation results with experimental data suggested the rodent specific site and distinct sitting-preferences of ligands on Template for human and rodent CYP1A1 enzymes. (3) Enhancement of metabolic activation and co-mutagenicity have been known as phenomena associated with Salmonella mutagenesis assay. Both the phenomena were examined on CYP-Templates in ways of simultaneous bi-molecule bindings of distinct ligands as trigger and pro-metabolized molecules. α-Naphthoflavone and norharman served consistently as trigger-molecules to support the oxidations of PAHs and arylamines sitting simultaneously as pro-metabolized molecules on Templates of CYP1A1, CYP1A2 and CYP3A4. These CYP-Template simulation systems with deciphering capabilities are promising tools to understand the mechanism basis of metabolic activations and to support confident judgements in safety assessments.

**Supplementary Information:**

The online version contains supplementary material available at 10.1186/s41021-023-00275-4.

## Introduction

Diverse types of chemicals act as carcinogens and genotoxic substances after their metabolic activations in the body. Cytochrome P450 (CYP) is associated in the metabolisms of these hydrophobic chemicals for both the detoxification and metabolic activation. Various CYP enzymes, expressed mainly in the liver and lesser extents in extra-hepatic tissues of humans, mediates oxidation and reduction of various chemicals including carcinogenic arenes and arylamines. Studies of crystalized CYP enzymes offered the 3D-structural models to predict metabolisms of ligands. The predictions of the regioselective metabolisms of ligands, particularly small-sized ones, are, however, not yet readily attained at present, possibly due to secondary interactions after ligand arrivals in the active site.

Studies of human CYPs associated with drug-metabolism are in unique situations. Experimental data on the catalytic properties of individual CYP enzymes have been accumulated in three-decades through the uses of their recombinant enzymes. With the use of these advantages, we have been developing *in silico* systems to understand CYP-mediated metabolisms by the ways of the reconstitutions of ligand-accessible spaces through assemblies of CYP ligands and also of the understanding of modes of interactions of CYP-residues with ligands in the active site. Starting from CYP2E1 [1], fused hexagonal-grid-based Templates^1^ were constructed for CYP2B6 [2], CYP2D6 [3] and CYP4A [4]. Template systems combined with ideas of ligand-interacting modes were established on CYP1A1 (> 350) [5], CYP1A2 (> 450) [6], CYP2C8 (> 360) [7], CYP2C9 (> 550) [8], CYP2C19 (> 450) [9], CYP2E1 (> 340) [10], CYP3A4 [11, 12], CYP3A5 [13] and CYP3A7 [13] (> 1,150 with CYP3As) through reciprocal comparison of simulation and experimental results (the numbers of reactions examined are shown in parentheses). Only ligand-drawing tools are necessary to perform these experiments. Placements of ligands on Template systems of human CYP1A1, CYP1A2, CYP2C8, CYP2C9, CYP2C19, CYP2E1 and CYP3As offered the information on sites of metabolisms regio- and stereo-selectively with more than 99% of accuracies. These Template systems are shown as effective tools for drug metabolism prediction and safety assessment [14–17].

In the present study, these fused grid-based CYP Template systems have been applied to three metabolism-associated phenomena of carcinogenic and/or mutagenic chemicals to decipher the underlying mechanisms. (1) CYP selectivity on metabolisms of typical CYP1A-inducers, (2) distinct catalytic properties of human and rodent CYP1A1 as well as CYP1A2, and (3) mechanisms of enhancement and co-mutagenicity.

## Materials and methods

### General properties of CYP templates

Experimental information on the substrate and inhibitor specificities of CYP enzymes and metabolisms of the substrates was obtained from literatures. The published data on recombinant CYP enzyme systems were used preferably because these data indicate the individual CYP enzyme properties distinctly. Chem3D (version 5 for Mac OS, CambridgeSoft, Cambridge, MA), ChemBio3D (version 12 for Windows, CambridgeSoft) and ChemBioDraw (versions 11 and 13 for Mac OS, CambridgeSoft/PerkinElmer) were used to construct two-dimensional (2D) and three-dimensional (3D) structures of the ligands and also to overlay compounds on Templates.

**Fig. 1:**
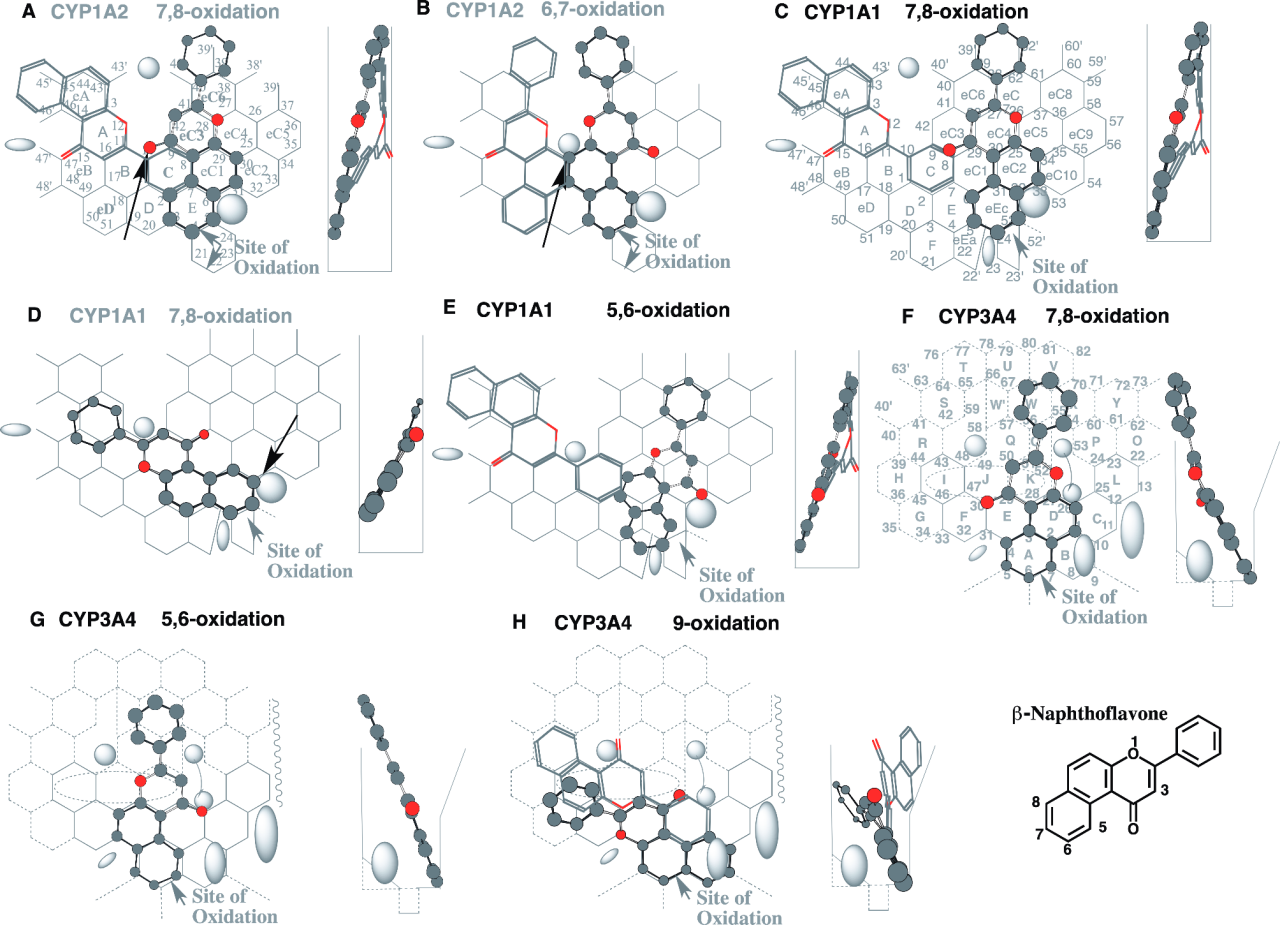
Simulated interactions of β-naphthoflavone with CYP1A2, CYP1A1 and CYP3A4. Placements of β-naphthoflavone for the 7,8- **(A)** and 6,7-oxidations **(B)** on CYP1A2 Template, for the 7,8- **(C** and **D)** and 5,6-oxidations **(E)** on CYP1A1 Template, and for the 7,8- **(F)**, 5,6- **(G)** and 9-oxidations **(H)** on CYP3A4 Template are shown as cylindrical shapes of 3D-structures for the pro-metabolized molecules and stick shapes of 3D-structures for trigger-molecules. In the right-side of each placement, 90°-rotated structures are indicated in Width-gauge. A 2D-structure of β-naphthoflavone is also shown with parts of position numbers. Functional and non-functional placements are indicated with dark- and grey-colored titles. Dark arrows indicate the possible causes of defects

**Fig. 2:**
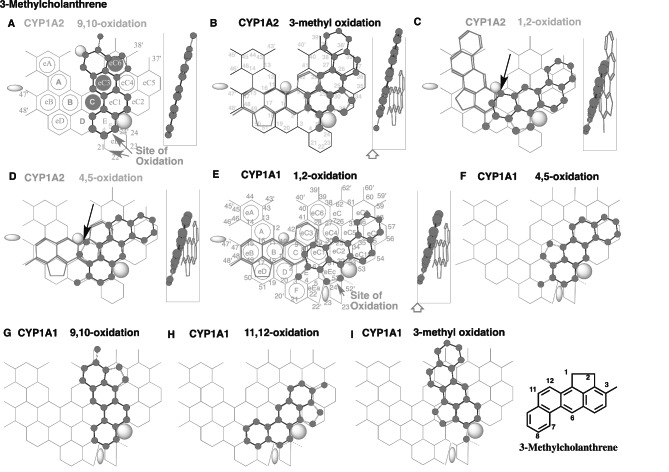
Simulated interactions of 3-methylcholanthrene with CYP1A2 and CYP1A1. Placements of 3-methylcholanthrene for the 9,10- **(A)**, 3-methyl **(B)**, 1,2- **(C)** and 4,5-oxidations **(D)** on CYP1A2 Template, and for the 1,2- **(E)**, 4,5- **(F)**, 9,10- **(G)**, 11,12- **(H)** and 3-methyl oxidations **(I)** on CYP1A1 Template are shown as cylindrical shapes of 3D-structures for the pro-metabolized molecules and as stick shapes of 3D-structures for the trigger-molecules. In the right-side of A-E, 90°-rotated structures are indicated in Width-gauge. Trigger molecules are omitted on F-I. A 2D-structure of 3-methylcholanthrene is also shown with parts of position numbers. Functional and non-functional placements are indicated with dark- and grey-colored titles. Dark arrows indicate the possible cause of defects

**Fig. 3:**
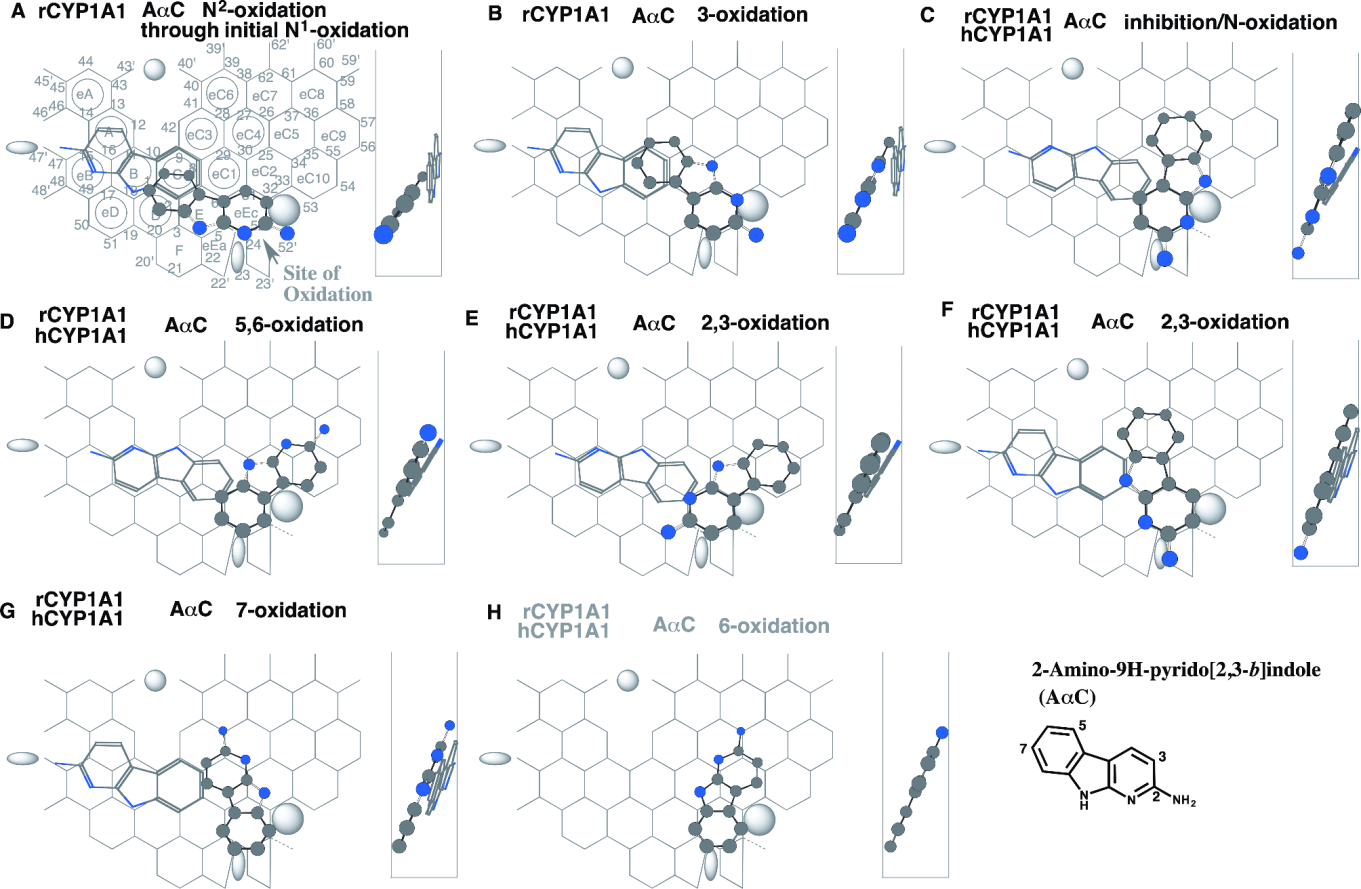
Interactions of 2-amino-9H-pyrido[2,3-*b*]indole (AαC) with CYP1A1. Placements of AαC for the *N*^2^- **(A)** and 3-oxidations **(B)**, inhibition/*N*-oxidation **(C)**, 5,6- **(D)**, 2,3- **(E** and **F)**, 7-oxidations **(G)**, and 6-oxidation **(H)** on CYP1A1 Template are shown as 3D-structures of cylindrical shapes for pro-metabolized molecules and of stick shapes for trigger molecules. In the right-side of each placement, 90°-rotated structures are indicated in Width-gauge. 2D-structures of AαC are also shown with parts of position numbers. hCYP1A1; human CYP1A1, rCYP1A1; rodent CYP1A1. Functional and non-functional placements are indicated with dark- and grey-colored titles. Dark arrows indicate the possible cause of defects

**Fig. 4:**
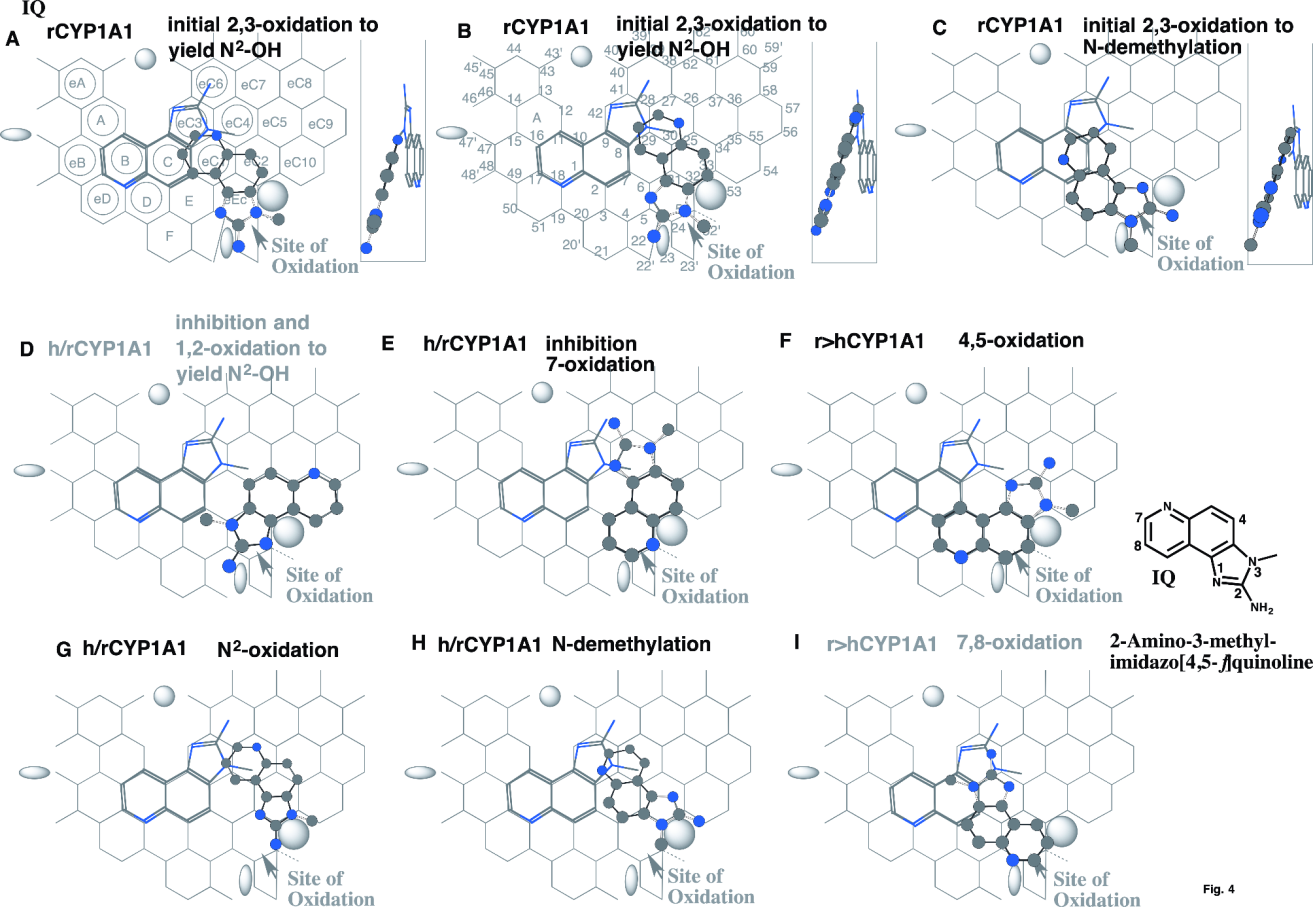
Interaction of 2-amino-3-methylimidazo[4,5-*f*]quinoline (IQ) with human and rodent CYP1A1. Placements of IQ for the *N*-oxidation **(A**, **B** and **G)**, *N*-demethylation (C and H), inhibition (D, E), 4,5- (F) and 7,8-oxidations **(I)** on CYP1A1 Template are shown as 3D-structures of cylindrical shapes for pro-metabolized molecules and of stick shapes for trigger molecules. In the right-side of A-C, 90°-rotated structures are indicated in Width-gauge. A 2D-structure of IQ is shown with parts of position numbers. hCYP1A1; human CYP1A1, rCYP1A1; rodent CYP1A1. Functional and non-functional placements are indicated with dark- and grey-colored titles

**Fig. 5:**
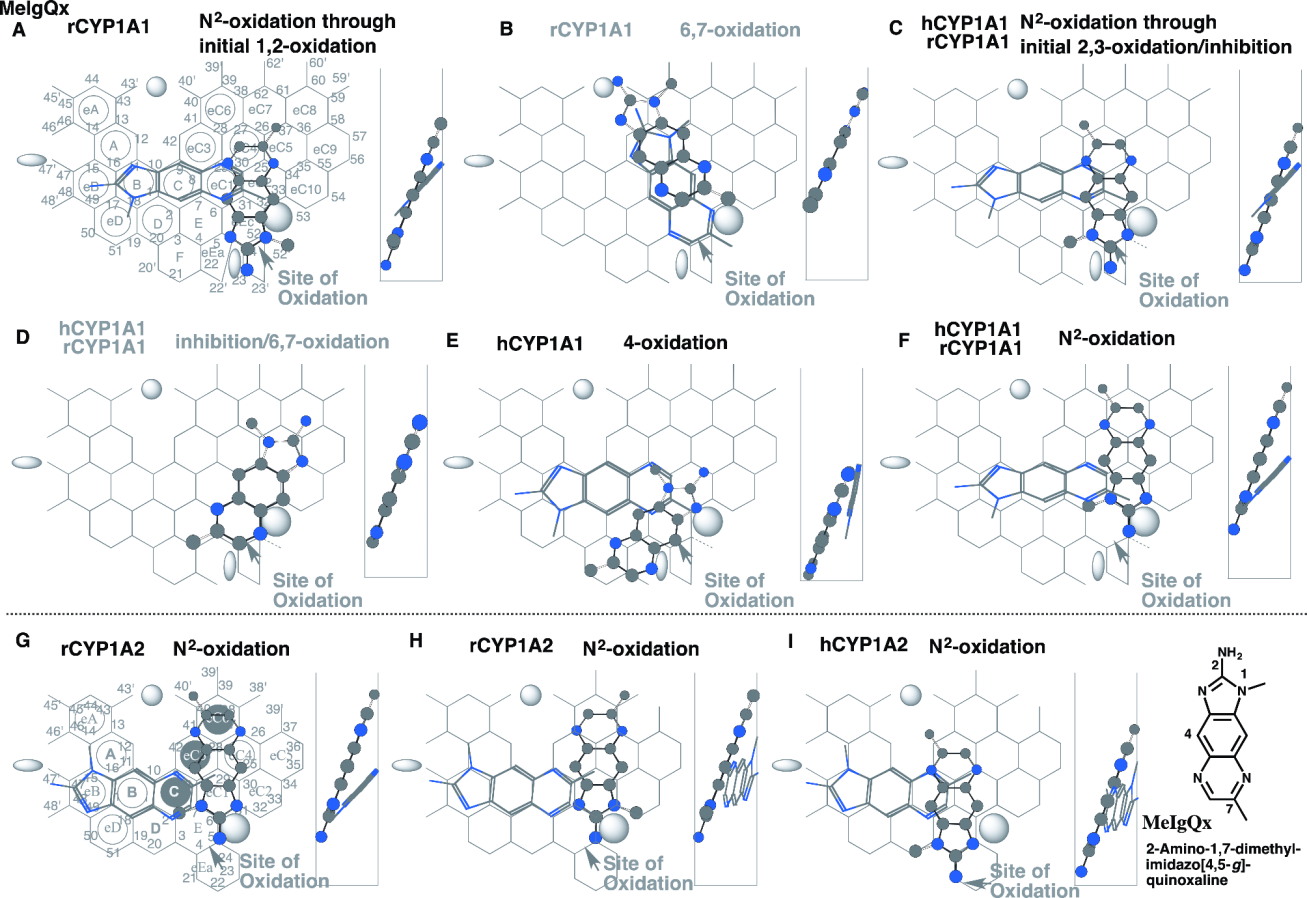
Interaction of 2-amino-1,7-dimethylimidazo[4,5-*g*]quinoxaline (MeIgQx) with human and rodent CYP1A1. Placements of MeIgQx for the *N*-oxidation **(A**, **C** and **F)**, 6,7-oxidation (B), inhibition (D), and 4-oxidation (E) on CYP1A1 Template, for the *N*-oxidation on CYP1A2 Template **(G**, **H** and **I)** are shown as 3D-structures of cylindrical shapes for pro-metabolized molecules and of stick shapes for trigger molecules. In the right-side, 90°-rotated structures are indicated in Width-gauge. A 2D-structure of MeIgQx is shown with parts of position numbers. hCYP1A1; human CYP1A1, rCYP1A1; rodent CYP1A1. Functional and non-functional placements are indicated with dark- and grey-colored titles

**Fig. 6:**
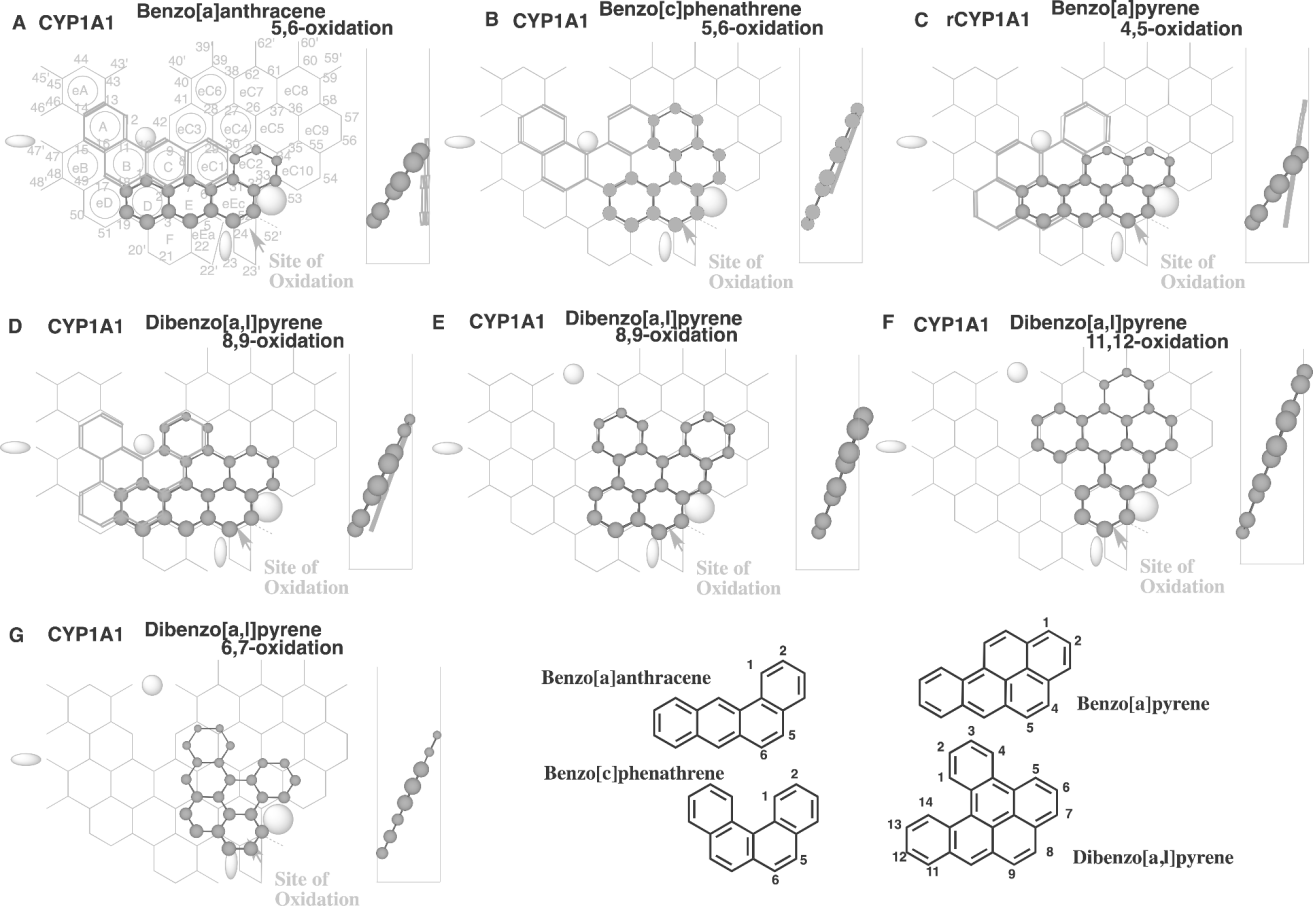
Interactions of typical PAHs with human and rodent CYP1A1. Placements of benzo[a]anthracene 5,6-oxidation, benzo[c]phenanthrene 5,6-oxidation, benzo[a]pyrene 4,5-oxidation, dibenzo[a,l]pyrene 8,9- **(D** and **E)**, 11,12- **(F)** and 6,7-oxidations (6G) on CYP1A1 Template are shown as 3D-structures of cylindrical shapes for pro-metabolized molecules and of stick shapes for trigger molecules. In the right-side, 90°-rotated structures are indicated in Width-gauge. Trigger-molecules are omitted on E-G. 2D-structures of ligands are shown with parts of position numbers

**Fig. 7:**
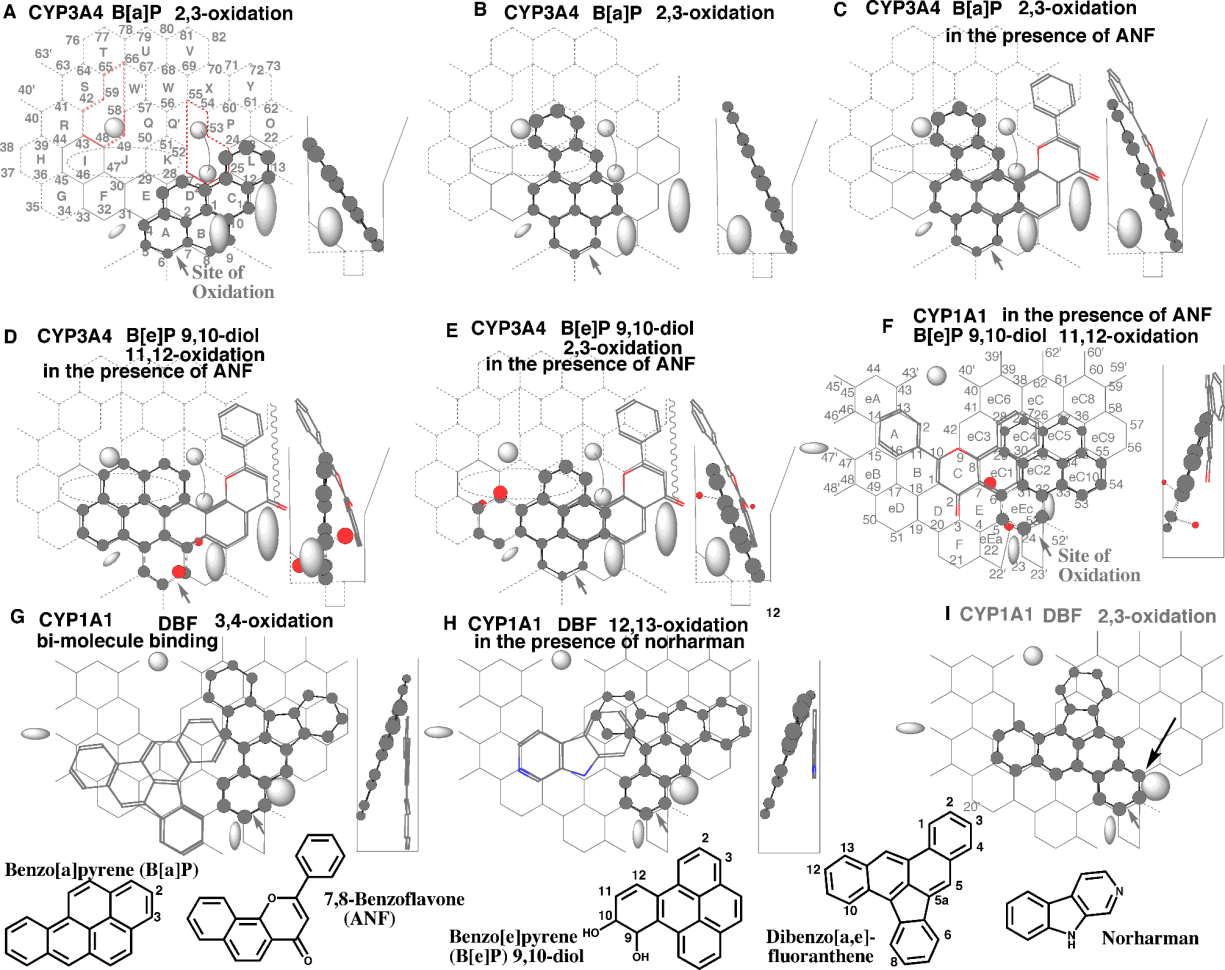
α-Naphthoflavone enhancement on CYP3A4 and norharman enhancement on CYP1A1. Placements of benzo[a]pyrene (BaP) 2,3-oxidation in the absence **(A** and **B)** and presence of α-naphthoflavone (ANF) **(C)**, of benzo[e]pyrene 9,10-dihydro-9,10-diol (BeP 9,10-diol) 11,12-oxidation **(D)** and 2,3-oxidation **(E)** on CYP3A4 Template, and the 11,12-oxidation **(F)** on CYP1A1 Template in the presence of ANF, and of dibenzo[a,e]fluoranthene (DBF) 3,4- **(G)** and 12,13-oxidations **(H)** in the presence of norharman and the 2,3-oxidation in the absence of norharman **(I)** are shown as 3D-structures of cylindrical shapes for pro-metabolized molecules and of stick shapes for trigger molecules. In the right-side, 90°-rotated structures are indicated in Width-gauge, except for I. Functional and non-functional placements are indicated with dark- and grey-colored titles. Dark arrows indicate the possible cause of defects. 2D-structures of ligands are shown with parts of position numbers in the bottom

**Fig. 8:**
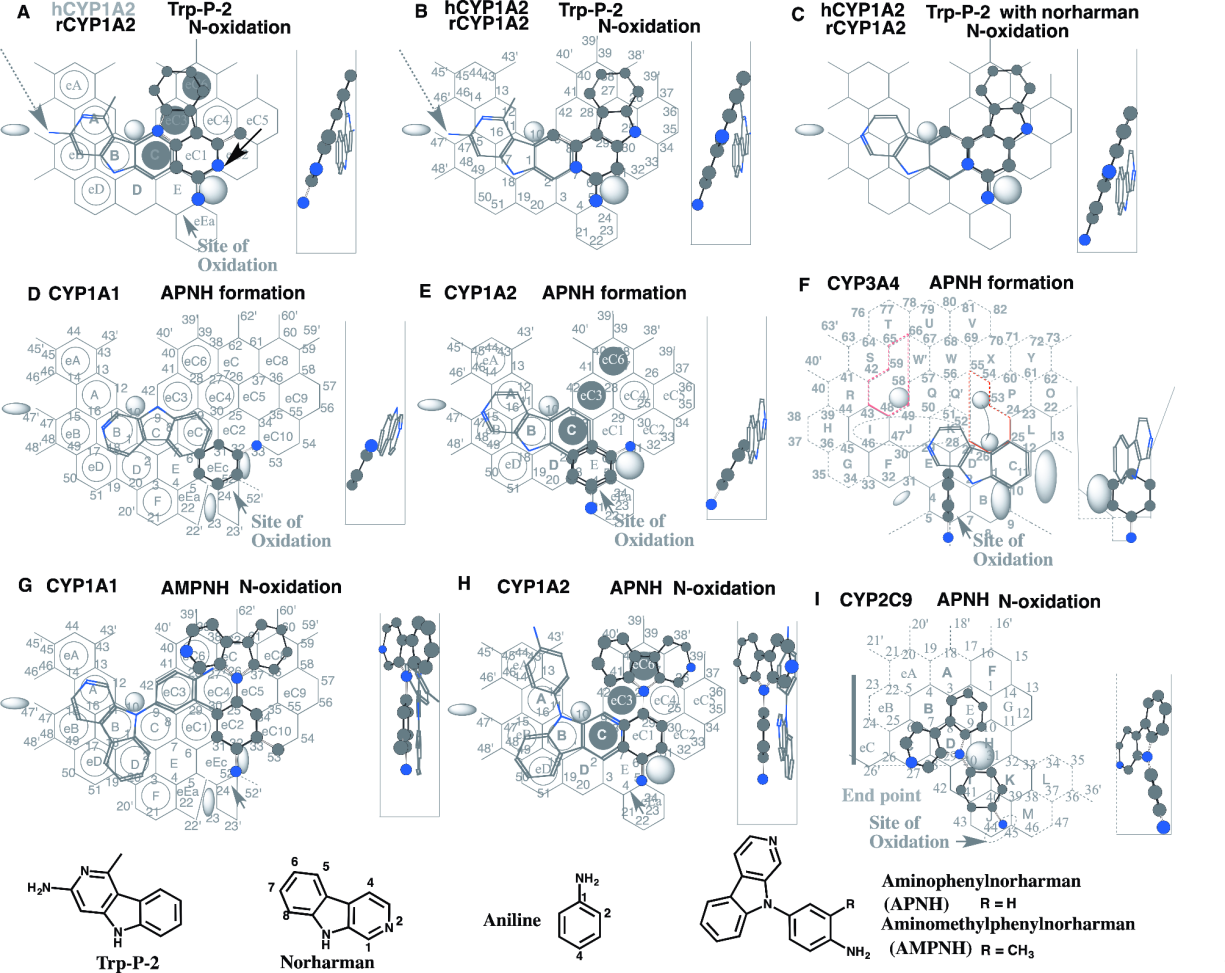
Norharman enhancements on CYP enzymes and co-mutagenicity Placements of Trp-P-2 *N*^2^-oxidation on CYP1A2 Template **(A** and **B)**, and in the presence of norharman **(C)**, of aniline for aminophenylnorharman (APNH) formation in the presence of norharman on Templates of CYP1A1 **(D)**, CYP1A2 **(E)** and CYP3A4 **(F)**, of the *N*-oxidation of aminomethylphenylnorharman (AMPNH) on CYP1A1 Template, and of the *N*-oxidations of APNH on Templates of CYP1A2 **(H)** and CYP2C9 **(I)** are shown as 3D-structures of cylindrical shapes for pro-metabolized molecules and of stick shapes for trigger molecules. In the right-side, 90°-rotated structures are indicated in Width-gauge. Grey-dashed and dark arrows indicate the possible causes of defects. and 2D-structures of ligands are shown with parts of position numbers in the bottom

Ligands of CYP enzymes, except for PAHs and arylamines, take various conformations due to their flexibility. Prior to the Template application, chemicals are taken in their flattened form(s). The flatted or extended shapes of 3D structures were tried to sit on Template and then modified their conformations to fit within Template in consideration of the bond-energy barrier using MM2 function of Chem3D and of specific interactions at distinct regions of Template. Carbon, oxygen, nitrogen, sulfur, fluorine and chlorine atoms of 3D ligand structures are indicated with grey, red, blue, yellow, khaki and green symbols, respectively (Figs. 1, 2, 3, 4, 5, 6, 7 and 8, Supplement Figs. 1, 2 and 3). The hydrogen atoms of the ligands were not considered for the placement. To avoid the confusion from stereo and Ring indications, italic symbols are used for chemical elements like *N* and *C* in the text, but not in figures.

Templates consist of hexagonal grids and sticks (Supplement Fig. 1). The sitting of ligand atoms at each corner of the hexagonal-grids (termed Rings) was evaluated as occupancy. Some atoms placed not exactly at the corner were accepted, if these atoms stayed within Template area or at specific defined sites. The placement of ligands is expressed in a hyphen-linked form, such as Rings A-B-C, to trace the occupancy of chemical molecules on Template. The branching part is indicated in the bracket. Ring names and Position numbers are assigned in ways to maintain their commonalities among Templates of CYP1A, CYP2C and CYP3A (Supplement Fig. 1). Ligands were assumed to migrate from Entrance to Site of oxidation without changing the conformation. Thus, ligands enter Template as the same conformations observed at Site of oxidation in general.

Ligands are necessary to interact with two-essential Positions, Site of oxidation and trigger-site, of CYP2E1 Template (Supplement Fig. 1E). An additional interaction to help the immobilization of ligand-sitting is necessary on CYP1A1- (Supplement Fig. 1A), CYP1A2- (Supplement Fig. 1C) and CYP3A4-Templates (Supplement Fig. 1F). Simultaneous plural-contact to Rear-wall is additionally required on CYP2C9- (Supplement Fig. 1G) and CYP2C19-Templates (Supplement Fig. 1H). Trigger-residues migrate after the arrival of ligands to Site of oxidation. These essential interactions are accomplished with single molecule (uni-molecule binding) or two molecules (bi-molecule binding) on Template. Idea of bi-molecule binding is introduced to explain the regioselective metabolisms on Template. In bi-molecule bindings, molecules sitting at Site of oxidation and immobilization-support part are termed pro-metabolized molecules. Second molecules, sitting at trigger-site, are termed trigger molecules. Pro-metabolized molecule and trigger molecule need to have a slight overlapping point on Template, although trigger molecules are situated behind to contribute for the immobilization of pro-metabolized molecules. Sitting of Trigger-residue thus initiates the catalysis at trigger site.

Chemicals including lactone moieties are often ionized at neutral pH ranges. These lactones were treated as ionizable groups for the application of substrates throughout CYP Template systems. Thus, non-rigid lactone rings are not allowed to contact with Rear-wall and with Trigger-residue in general.

Allowable depths of ligands are indicated as Width-gauge. Distance between Facial and Rear walls of Width-gauge differ depending on the enzymes.

Several template-terms are defined to explain ligand interactions with Template. These terms are listed in a separate section as terms used for Template system (Table 1).

**Table 1:** Terms used for Template system

**2D and 3D:** two-dimensional and three-dimensional
**Bay 1 and Bay 2:** Residues located lower left and right of CYP1A, CYP2C and CYP3A Templates
**Bi-molecule and uni-molecule binding:** Interactions on Template with Trigger- and Pro-metabolized molecules combination, and with single molecule
**Cavity-1 and Cavity-2:** Holes in the middle of CYP3A4 Template. The residues in the holes (**Cavity-1 residue and Cavity-2 residue**) are expected to participate in the IJK-Interaction and triggering. These residues appear on Template plane after ligand’s passage
**Entrance:** Routes for the entry of ligands
**Facial-side movement:** CYP1A2-mediated pushing at Ring C, to transfer substrates toward Site of oxidation
**Fjord:** A central space at where no substrate stays on CYP1A Template
**Front-residue:** A residue existing at facial side of Ring B of CYP3A Template
**Futile-sitting:** A phenomenon of lack of oxidations associated with rotatable and non-substituted phenyl group of ligands
**Groove:** A space for ligand sittings located beneath of Width-gauge of CYP3A and CYP2E1
**IJK-Interaction:** Interaction of ligands with Rings I, J and/or K region is expected to initiate facial-side movement of ligand on CYP3A Template
**Left-end:** The left-side border of CYP2C Template located between Entrance and Shelf
**N-Atom impedance:** Rejection of sitting of pyridyl or secondary amino nitrogen atom at Position 31 of Ring eC1 of human CYP1A2
**Placement Type:** There are seven distinct types of ligand sittings on CYP3A4 Template, termed Type-1 to Type-7. These are associated with the usage frequencies for the interaction
**Preferred order:** Occurrence order of the reaction determined by the placement on CYP1A Template
**Pro-metabolized molecule:** Substrates to be oxidized or reduced are termed as “pro-metabolized molecule” in the simulation experiment
**Rear-residue:** A residue sitting at rear-side of Position 30 of CYP3A7 Template
**Right-side movement:** Right-direction shift of ligands entered in Rings A and B to Bay-2 direction on CYP3A4 Template
**Shelf:** A plateau-like shape area between Position 29 and Left-end of CYP2C Template
**Sideway:** Ligand migration route in the right side of Center-Area, which corresponds to Rings E-eEc-eC2-eC10-eC5-eC9-eC8 on CYP1A1 Template
**Simultaneous plural-point contact:** Initial interactions of CYP2C ligands start with their simultaneous plural-parts contact to Rear-wall standing upright at rear end of Template
**Site of oxidation:** A confined space of enzymatic catalysis to interact with heme
**Thick-area:** An area of CYP1A2 Template on where flexible or bulky shapes of chemicals as well as PAHs interact
**Thin-area:** A flat-area of CYP1A2 where flat shapes of aromatic hydrocarbons stay
**Trigger molecule:** A molecule, which is not oxidized, acts for triggering the catalysis. Trigger molecules need to have an overlap with pro-metabolized molecules on Template
**Trigger-site:** A site which Trigger residue moves to hold ligands to initiate catalyses. For examples, Position 26 of CYP3A Template, Position 10–11 of CYP1A and Position 11 of CYP2E1 correspond to Trigger-site
**Type-1, Type-2 and Type-3 placements on CYP1A1:** Ligands hang on Bay 2 residue through sitting at Position 33 of Ring eC2 on Type-1 placement. Ligands contact with Bay-2 residue at Ring eEc and have a protruding part on Ring eEa on Type-2 placement. Ligands take bottom-flattened sittings contacting simultaneously with the side part to Bay 2 residue and with the flat part to Bottom-residue on Type-3 placement
**Uni-molecule and bi-molecule binding:** Placements on Template with single molecule and with Trigger and Pro-metabolized molecules, respectively
**Width-gauge:** A guide tool to verify allowable width for ligand accommodation around Template which was determined empirically

### Description on specific CYP template

#### CYP1A1

CYP1A1 Template has Entrance-1 and Entrance-2 for the ligand (Supplement Fig. 1A). A space of Rings eC1-eC10 is termed Thin-area and a space of Rings A-E is termed Thick-area having relatively thick-space on the Template [5]. Thin-shape chemicals like PAHs thus stay more on Thin-area linking to Entrance-2. These ligands are fastened at Center-spot or Sideway area [5]. Near the Site of oxidation, ligands of CYP1A1 are necessary to contact Bay-2 residue at Positions 32 and 52 of Ring eEc (Bay-2 interaction). There are three common ways of Bay 2 interaction to sit at Site of oxidation. 1. Ligands hang on Bay 2 residue through sitting at Position 33 of Ring eC2 (Type-1 placement, See Fig. 3D). 2. Ligands contact with Bay-2 residue at Ring eEc and have a protruding part on Ring eEa (Type-2 placement, See Fig. 3F). 3. Bottom-flattened ligands contact simultaneously with the side part to Bay 2 residue and with the flat part to Bottom-residue (Type-3 placement,　See Fig. 3G). Ligands also take two other placements on CYP1A1 Template. One of the placements having Pier-sitting (Position 52’) is precedent than all the others and only available on rodent CYP1A1. The other is a placement selective for linear three-ring arenes like dibenzo-*p*-dioxin (oxanthrene) sitting on Rings eEa-eEc-eC2 (See Supplement Fig. 3G). No contact with Center-spot is required for both types of placements. Both placements are precedent to Type-1, Type-2 and Type-3 placements, if available.

Most common Type-1 placement is hanging at Position 33 and occupying fully Ring eEc. Within the Type-1 sittings, placements using Sideway (area of Rings eEc-eC2-eC10-eC9) precede placements having Center-spot contact on human CYP1A1 Template. Additional sitting at Position 23 like Type-2 sitting offers the advantage for the rigid contact on CYP1A1 Template.

Placements using Ring D and/or E sitting may share with relative high frequencies on rodent CYP1A1 Template than on human CYP1A1 Template.

Type-2 placements (a protruding part at Position 23) occupying fully Ring eEc and Center-spot, and then Type-3 placements with Center-spot contact are followed. Sittings occupying partially Ring eEc may also contribute, unless the placements described above are available.

All the ligand placements are necessary to occupy Position 10 for trigger-action in uni-molecule or bi-molecule bindings. Width-gauge is introduced in the renewed CYP1A1 system (Supplement Fig. 1A). Distance between Facial- and Rear-walls of Width-gauge was determined arbitrarily as 1.5 Ring diameter from the simulation results of several CYP1A1 ligands. Trigger molecules are allowed to sit on Rings eA, A, eB, B, C, eD, D, F, eC1, eC3, eC4, and eC6 (Trigger-molecule harboring area shown as circle symbols).

CYP1A1 catalyzes oxidations of arylalkylates as well as PAHs. For example, human CYP1A1 mediates an intramolecular rearrangement of the aminoazepane moiety of GDC-0339 [18]. A placement for the pyrazole oxidation to link to the rearrangement was generated at Rings eA-A-eB-B(eD)-D-C-E-eEc-eC1-eC4-eC6 plus a space at Bay-1 (Supplement Fig. 1B). This molecule fulfilled three essential interactions at Site of oxidation (Positions 24/52), trigger-site (Positions 10/11) and Center-spot (Positions 27/28) as uni-molecule binding. The GDC-0339 molecule was expected to migrate to the left-direction from Entrance-2 to Ring A judged from the molecule size and gate sizes between Fjord/Bay-1 residues and Fjord/Bay-2 residues. The bulky aminoazepane, rather than the aryl moiety, sat on Thin-area of CYP1A1 Template and stayed within Width-gauge.

#### CYP1A2

Ligands enter CYP1A2 Template through Entrance-1 and Entrance-2. Entrance-1 links to a relatively thick space (Rings A-E) and Entrance-2 to a thin-space (Rings eC1-eC6) (Supplement Fig. 1C). PAHs are thus more often fastened on the thin-space (Thin-area), while flexible alkylates sit on the thick space (Thick-area) [19]. All the ligands are necessary to occupy Positions 10–11 for trigger-action in uni-molecule or bi-molecule bindings. Occupancy of ligands at Position 9 on Thick-area is also required for Facial-side movement [20]. Trigger molecules are allowed to sit on Rings eA, A, eB, B, C, eD, D, eC3, eC4, and eC6 (Trigger-molecule harboring area shown as open and closed circle symbols). Closed circle symbols indicate the region allowing the overlap of pro-metabolized and trigger molecules.

Width-gauge was introduced in the renewed CYP1A2 system [17]. Distance between Facial- and Rear-walls of Width-gauge was determined arbitrarily as 1.5 Ring diameter from the simulation results of several CYP1A2 ligands (Supplement Fig. 1C).

Reciprocal comparison of simulation results of ligands with experimental data suggested distinct uses of Template area as the causes of different regioselectivities between human CYP1A2 and rodent CYP1A2 [6]. For example, rodent CYP1A2, but not human CYP1A2, mediates 8,9-oxidation of dibenzo[a,l]pyrene [21]. A placement for the 8,9-oxidation was constructed at Rings D-C(E)-eC1-eC4 plus a space at Fjord (Supplement Fig. 1D). The molecule needed the migration from Entrance-2 to Ring D, which was only allowed on rodent CYP1A2. In addition, the molecule leaning against Rear-wall was able to contact with descended Trigger (Fjord)-residue around Position 10 on Template. The placement of dibenzo[a,l]pyrene 8,9-oxidation also suggested the descending of Trigger-residue around center to rear-side of Width-gauge (Supplement Fig. 1D right). Distinct uses of Ring eEa and rejection of a basic nitrogen atom at Position 31 (N-atom impedance) also contribute on the differences of regioselectivities of human CYP1A2 and rodent CYP1A2 [6].

#### CYP2E1

Ligands migrate from the top of CYP2E1 Template (Supplement Fig. 1E) and need to pass a gate between the left-end of Ring A (Positions 16/17) and Trigger-residue (Position 11/12). Trigger-residue moves from Position 11/12 to Positions 1/14 after the contacts of ligands at Site of oxidation, if possible. A groove is located beneath of Position 4 for ligand sitting. Facial- and Rear-walls of CYP2E1 Template was not parallel, narrow in the left-side and wide in the right-side. Only aryl structures are accepted on Rings eA, A and C. Maximal depth of Width-gauge at the right-end (Shown as right-side of supplement Fig. 1E) and size of Trigger-residue are determined arbitrary as one- and one-third of Ring diameter, respectively [10]. Often bi-molecule bindings are necessary for CYP2E1-mediated oxidations of tri- and tetra-arenes like phenanthrene and benzo[c]phenanthrene [10]. Trigger-residue interacted with pro-metabolized molecules of these ligands and moved their locations. Other experimental procedures are described in our publications [1, 10].

#### CYP3A4

Ligands enter from the left-side and migrate to Site of oxidation (Position 6/7) at the right-side bottom of CYP3A4 Template (Supplement Fig. 1F) [12]. The molecules are necessary to occupy at least a part of Rings I-J-K region for I-J-K-Interaction. Maximal thickness of ligand space is set from sittings of various steroid ligands as Width-gauge [22]. There are two regions excluding the sitting of ligands at the middle of Template, termed Cavity-1 and Cavity-2 (Supplement Fig. 1F). Cavity-1 residue appeared on Template after the passage of ligands supports the immobilization of ligands. Cavity-2 (Trigger) residue stays initially around Position 53 and near Rear-wall, and then slides down to Position 26 after the arrival of ligands at Site of oxidation. Presence at the initial Position of Cavity-2 residue sometimes interferes the ligand migrations like the cases of clarithromycin [12] and ebastine [15], and restricts the migration of ligands beyond trigger-site (Position 26). Ligands are thus able to pass a space around Facial-wall, but not a space center to Rear-wall, around Cavity-2 residue. Detailed procedures are described in our publications [12–15, 22, 23].

#### CYP2C9, CYP2C19 and CYP2C8

Ligands of both CYP2C9 and CYP2C19 go down from top to the Site of oxidation at a bottom part (Supplement Fig. 1G and H). Most of the CYP2C ligands sit on Shelf to interact with Trigger-residue. These ligands are fastened with the aids of the interaction at Position 29 and Left-end. Trigger-residues of CYP2C9 and CYP2C19 move to the left direction near to Position 28 (CYP2C9) and to the middle of Positions 29 and 30 (CYP2C19) to immobilized the ligands.

Procedures for applying ligands to CYP2C8 Template are described in our recent publication [7].

## Results

### CYP selectivity on metabolisms of typical CYP1A-inducers

On Salmonella mutagen-test system, 9,000 g supernatants, so called S9, are an essential component to detect pro-mutagens. S9 fraction prepared from livers of rodents pretreated with polychlorinated biphenyl mixtures like Aroclor 1254 or phenobarbital/3-methylcholanthrene mixture had been used in the assay system. These agents were used as inducers to enhance the S9 capacities for metabolic activations, and substituted currently to phenobarbital/β-naphthoflavone(5,6-benzoflavone) mixture. β-Naphthoflavone and 3-methylcholanthrene are ligands of Ah-receptor, which regulate transcriptional activation of a group of drug-metabolizing enzymes including CYP1A1 and CYP1A2. There are many PAHs having high affinities to Ah receptor, but β-naphthoflavone and historically 3-methylcholanthrene have been selectively used for liver homogenates or the S9 preparations due to the efficient induction of drug metabolizing enzymes [24]. The metabolic clearance in the body, in addition to the high affinities to the transcriptional factors [25, 26], is considered to be a factor for the efficient inductions after β-naphthoflavone and 3-methylcholanthrene treatments. Involvement of P-448 type enzymes is known on the metabolism of both chemicals [27, 28]. No rigid information at the levels of CYP enzymes is, however, available until now. To verify the roles of CYP1A1 and CYP1A2 on the metabolism of β-naphthoflavone and 3-methylcholanthrene and the relationship with the efficient enzyme induction, oxidative metabolisms of both chemicals were assessed on CYP1A1 and CYP1A2 Templates.

#### β-Naphthoflavone on CYP templates

β-Naphthoflavone (5,6-benzoflavone) undergoes the 7,8- and 5,6-oxidations in liver microsomes prepared from 3-methylcholanthrene-treated rats [28]. To verify the role of individual CYP enzymes, metabolisms were investigated *in silico* using fused-grid based CYP-Template systems. At first, β-naphthoflavone molecule was applied on CYP1A2 Template [6, 17].

A placement for the 7,8-oxidation of β-naphthoflavone was generated as bi-molecule bindings on CYP1A2 Template at Rings E-eC1-eC3(C)-eC6 plus a space above Ring eC6 for the pro-metabolized molecule and at Rings C-B-A-eA plus Position 47 and a space left-side of Ring eA for the trigger molecule (Fig. 1A) or at Rings eD-B-A(eB)-eA plus a space above Ring eA for the trigger molecule (Data not shown). The trigger molecule is necessary to support the trigger-interaction at Positions 10–11 to hold Trigger (Fjord)-residue descended and immobilization of the pro-metabolized molecule on CYP1A2 Template system [19]. Trigger-molecule has to pass a gate between Bay-1 and Trigger (Fjord)-residues to enter CYP1A2 Template area. The pro-metabolized molecule occupied Position 10 on the β-naphthoflavone placement (Fig. 1A), but the occupancy was not enough support to hold Trigger (Fjord)-residue descended. The trigger molecule might thus contact with the pro-metabolized molecule. The descending of Trigger (Fjord)-residue was, however, expected to break the contact of the ligands at Trigger-site (Positions 10 and 11) on CYP1A2 Template. Overlaps of pro-metabolized and trigger molecules at Trigger-site (Positions 10 and 11) thus resulted in the collapse of the bi-molecule placement of β-naphthoflavone on CYP1A2 Template system.

A distinct placement for the 6,7-oxidation was constructed at Rings E-C-eC3(eC4)-eC6 plus a space above Ring eC6 for the pro-metabolized molecule (Fig. 1B). The placement might also have the contact with the trigger-molecule at Position 10, and was expected to be non-functional through Trigger (Fjord)-residue-mediated breaking of the pro-metabolized molecule placement.

Both the placements on CYP1A2 Template suggested the scare catalyses of CYP1A2 for the oxidation of β-naphthoflavone through the indecisive bindings of the pro-metabolized molecule at Trigger-site (Position 10).

CYP1A1, as well as CYP1A2, become major hemoproteins in livers after treatments of rats with PAHs. None or only in trace levels of CYP1A1 is, however, detected in livers of untreated animals and of normal human individuals [29–31]. β-Naphthoflavone molecule was applied on CYP1A1 Template to assess the role of this enzyme [5]. A sitting for the 7,8-oxidation was available at Ring eEc-eC2-eC4(eC1)-eC7 plus a space above Rings eC6 and eC7 as the pro-metabolized molecule as Type-1 placement, and at Rings eA-A-B-C plus Position 47 and a space left side of Ring eA as the trigger-molecule (Fig. 1C). Although a contact of the pro-metabolized and trigger molecules was limited at Position 8, the Type-1 placement had a hanging at Position 33, full Ring eEc occupancy and Center-spot (Positions 27/28) contact. A placement of uni-molecule binding was constructed for the 7,8-oxidation at Rings eEc-E-C-B-A plus Position 29 (Fig. 1D). No hanging at Position 33 was available on this placement and thus this placement was judged to be non-functional.

A distinct Type-3 placement having flat-bottom interaction with Bay-2 was generated for the 5,6-oxidation at Rings eEa-E-eEc-eC1-eC2-eC5-eC7(eC8) as the pro-metabolized molecule (Fig. 1E). The molecule was hooked with the carbonyl part around Position 33. Both the pro-metabolized molecules for the 7,8- (Fig. 1A) and 5,6-oxidations (Fig. 1E) contacted with the trigger-molecules at Positions other than Trigger-site (Positions 10–11). These results suggested the functional contribution of CYP1A1 for both the 7,8- and 5,6-oxidations of β-naphthoflavone on Template.

These results were consistent with the data of β-naphthoflavone oxidation mediated by purified rat-P450c corresponding to rat CYP1A1 [28].

Low levels of the 5,6- and 7,8-oxidation products and a phenolic metabolite of β-naphthoflavone were detected in liver microsomes of untreated and phenobarbital-treated rats [28]. Hepatic levels of phenobarbital-inducible P450b and P450e, corresponding respectively to CYP2B1and CYP2B2, were very low in the liver of untreated rats [32]. An alternate role of CYP3A enzyme was thus tested with the use of CYP3A4 Template. Three placements of β-naphthoflavone were generated as uni-molecule bindings at Rings A-D-K(J)-Q’-W(X) for 7,8-oxidation (Fig. 1F), and at Rings A-E-K-Q’-W plus Position 26 (Trigger-site) for the initial 5,6-oxidations to yield the 5-hydroxy metabolites (Fig. 1G). A bi-molecule binding for the 9-oxidation was constructed at Rings B-A-E(K)-F-G(I) for the pro-metabolized molecule and at Rings D-E-J-I-R plus Position 58 for the trigger molecule (Fig. 1H). The 2-phenyl part at Position 26 of Ring D might be not fastened well and thus suggested the inefficient oxidation. A metabolite (Phenol-A) [28] might be corresponded to 9-hydroxy-β-naphthoflavone, if possible. Placements of β-naphthoflavone 9-oxidation were also generated on CYP2C9 Template at Rings M-J-I-D-C plus Position 32 and on CYP2C19 Template at Rings K-I-D-C-eC plus Position 10, respectively (Data not shown). These results might suggest the slight contributions of CYP2C and CYP3A enzymes on the oxidations of β-naphthoflavone in livers.

#### 3-Methylcholanthrene on CYP Template

Metabolisms of 3-methylcholanthrene were studied in 60’ to 80’ to understand the metabolic fate in rats, but remained yet clarified for the role of the exact CYP enzyme involved [33, 34]. 3-Methylcholanthrene undergoes the oxidations mainly at the 1- and 2-positions, 9,10-bond and 11,12-bond [33–35], and also the 3-methyl part [36, 37].

Among the placements of 3-methylcholanthrene on CYP1A2 Template, A most preferred placement, at Rings E-eC1-eC3-eC6 plus a space left-side above Ring eC3 in Fjord area, was constructed for the 9,10-oxidation (Fig. 2A). The ligand part at Fjord area would, however, contact with Trigger-residue and thus interfere with the descending of Trigger-residue. A second-preferred placement was at Rings E-eC1(eC2)-eC4-eC7 plus a space above Rings eC6 and eC7 for the pro-metabolized molecule of the 3-methyl oxidation (Fig. 2B). Additional placements were constructed at Rings D-C(E)-eC1-eC2-eC5(eC4) for the pro-metabolized molecule of the 1,2-oxidation (Fig. 2C) and at Rings D-E-C-eC1-eC4-eC5 for the pro-metabolized molecule of the 4,5-oxidation (Fig. 2D). These placements using Ring D are possible only on rodent CYP1A2 Template, but not substantially on human CYP1A2 Template due to the difficulties to use Ring D [6]. Both the pro-metabolized molecule placements for the 1,2- and 4,5-oxidations occupied around Trigger-site (Positions 10–11), but not enough for supporting trigger-action. Introduction of the trigger molecules would, however, result in the collapse of the bi-molecule bindings at the descending of Trigger-residue, in similar to the case with CYP1A2-mediated oxidation of β-naphthoflavone described above (Fig. 1A and B). These simulation data suggested the poor CYP1A2-mediated oxidation of 3-methylcholanthre, except for the 3-methyl oxidation.

The interference of Trigger-residue descending, in the most preferred placement for the 9,10-oxidation (Fig. 2A), was possible to impair/diminish the total catalyses of CYP1A2 for 3-methylchlanthrene oxidations. Inhibitions through Trigger-residue interactions are also expected with other chemicals [17].

Pro-metabolized molecule placements on CYP1A1 Template for the 1,2-, 4,5-, 9,10-, 11,12-oxidations, and 3-methyl oxidation of 3-methylcholanthrene were available at Rings eEc-eC1(E)-eC2-eC10-eC9(eC5) (Fig. 2E), at Rings eEc(E)-eC1-eC2-eC5-eC9 (Fig. 2F), at Rings eEc-eC2-eC4-eC6(eC7) plus Position 39’ (Fig. 2G), at Rings E-eEc-eC2(eC10)-eC5-eC9 (Fig. 2H), and at Ring eEc-eC2(eC1)-eC4-eC6 plus a space above Rings eC6 and eC7 (Fig. 2I), respectively. Their trigger molecules were generated at Rings eB(eD)-B-C-eC3 plus Position 48’of the Template (Fig. 2E). CYP1A1 was also expected to mediate 11,12-epoxidation of 3-methylcholanthrene 9,10-dihydrodiol from the placement similar to that of the 11,12-oxidation of 3-methylcholanthrene (Fig. 2H).

Experimental data of the oxidative metabolism of 3-methylcholanthrene in hepatic preparation of untreated animals is limited [35]. Possible involvements of constitutive CYP3A and CYP2C enzymes on 3-methylcholanthrene oxidations were thus examined using CYP3A4 and CYP2C9 Templates. Placements for the 1, 2- and 9, 10-oxidations and 3-methyl oxidation were available at Rings A(D-B)-E-F-I(G), at Rings A-D-K-Q-W plus facial-side of Cavity (Trigger)-2 residue, and at Rings A-D-E-K-Q-W on CYP3A4 Template (Data not shown). All the three interactions with Trigger-residue at Position 26 were labile. CYP3A4 was thus expected to contribute slightly at high levels of the exposure. These results further supported the view of the dominant role of CYP1A1 on oxidative metabolisms of 3-methylcholanthrene.

### Distinct catalytic properties of human and rodent CYP1A1 as well as CYP1A2

A common name CYP1A1 is used for both the rodent (rat and mouse) and human enzymes. Differences are, however, known on their catalytic properties toward several chemicals including PCB126 (3,3′,4,4′,5-pentachlorobiphenyl) [38], aristolochic acid [39] and dioxins [40]. Simulation experiments of these ligands suggested the different usages in two sites (Pier and Ring F/D sittings) on CYP1A1 Template, which linked with distinct catalyses of human CYP1A1 and rodent CYP1A1 [5]. For example, sittings of an extra space at Position 52’ (Pier-sitting) occurred on rodent CYP1A1-mediated oxidation of PCB126. Both uni-molecule and bi-molecule bindings were generated for 4-hydroxy-3,3’,4’,5-tetrachlorobiphenyl formation at Rings eEc-eC1-C-B plus Positions 23, 52’ and 33 (Supplement Fig. 3A) and at Rings eEc-eC1-eC4-eC7 plus Positions 4, 23 and 52’ for the pro-metabolized molecule (Supplement Fig. 3B). A minor metabolite, 4-hydroxy-3,3’,4’,5,5’-pentachlorobiphenyl, was expected from the uni-molecule placement at Rings eEc-eC1-C-B plus Positions 33, 42 and 52’ (Supplement Fig. 3C). The bulky chlorine atoms at Position 11 and 42 might reduce the chance of Fjord (Trigger)-residue to reach to Trigger-site.

A flavanone, naringenin, inhibited strikingly rat CYP1A1-mediated (Ki value 0.17 μM), but only faintly human CYP1A1-mediated (Ki value 489 μM), *O*-deethylation of 7-*O*-ethylresorufin [41].

Both uni-molecule and bi-molecule bindings of naringenin were generated on CYP1A1 Template. The uni-molecule placement at Rings eEc-E-C-B-A plus Positions 22, 23 and 33 (Supplement Fig. 3D) was, however, unstable on Fjord (Trigger)-residue interaction and thus unlikely to function. The bi-molecule placement was available at ring eEc(E)-eC2-(eC10)-eC4-eC7 plus Position 52’ and a space near Position 62’ as the pro-metabolized molecule (Supplement Fig. 3E). The flipping of this molecule also generated a bi-molecule placement having Pier-sitting at Rings eEc-eC1-eC4-eC6 plus Positions 2, 4 and 52’ and around Position 39’ (Data not shown). These molecules sitting at Position 52’ were expected to work for the inhibitory action. The former sandwiched Bay-2 residue for the tight interaction, and the latter might migrate from Entrance-1 relatively easier than the former. No metabolism of [^3^H-]naringenin was detected with the lymphoblastoid microsomes containing individually expressed human CYP1A1 [42], suggesting the substantial lack of human CYP1A1-naringenin interaction.

These data supported the idea of Pier-sitting of *C*1-equivalent of ligands selectively on rodent CYP1A1.

#### Differences on 2-amino-9H-pyrido[2,3-*b*]indole (AαC)

Clear differences between human CYP1A1 and rat CYP1A1 are known on the oxidations of heterocyclic arylamines, 2-amino-9H-pyrido[2,3-*b*]indole (AαC) and 2-amino-3-methyl-9H-pyrido[2,3-*b*]indole (MeAαC). Rat CYP1A1, but not human CYP1A1, metabolizes extensively both the amines [43]. Both enzymes also differ in regioselectivity. Rat CYP1A1 mediates the *N*^2^-, 6-, 3- and unidentified sites of AαC oxidations in the decreasing order, while human CYP1A1 oxidizes AαC at the 6-, 3- and unidentified sites in the decreasing order.

Substrates of CYP1A1 are necessary to occupy simultaneously both Trigger-site and Site of oxidation on CYP1A1 Template. Two-molecules of AαC are thus necessary to cover as bi-molecule binding. Following description was focused on the pro-metabolized molecule of AαC.

Plural placements of AαC using Pier-sitting were generated on rodent CYP1A1 Template at Rings eEc-E-C(D) plus Position 52’ for the initial *N*^1^-oxidation leading to the *N*^2^-hydroxylamine formation (Fig. 3A), and at Rings eEc-eC1-eC3(C) plus Position 52’ for the 2,3-oxidation (Fig. 3B). No occupancy at Positions 27 and 28 was necessary for placements using Pier-sitting. These molecules interacted with trigger-molecule sitting at Rings A(eB)-B-C. The pro-metabolized molecule for the *N*-oxidation was judged to be the primary placement. The molecule for the 2,3-oxidation was expected to be the second-preferred placements of AαC on rodent CYP1A1 Template, although Trigger-residue might break partly the bi-molecule binding. The third-preferred placement for rodent CYP1A1 was generated at Rings eEc-eC2-eC4(eC5) plus Position 23 (Fig. 3C), which hanged on Bay 2 residue through sitting at Position 33 of Ring eC2 (Type-1 interaction) and also protruded on Ring eEa (Type-2 interaction). This placement corresponded to the primary placement on human CYP1A1, and suggested the inhibition through the interaction with the *N*^1^-part of AαC for both human and rodent CYP1A1 enzymes. Two Type-1 sittings of the pro-metabolized molecules were available at Rings eEc-eC2-eC10(eC5)-eC9 for the 5,6-oxidation (Fig. 3D) and at Rings eEc(E)-eC2-eC10(eC5)-eC9 for the 3,4-oxidation (Fig. 3E). Both the Type-1 sittings took Sideway-route to migrate to the Site of oxidation of CYP1A1 Template, and were thus not required the occupancies at Positions 27 and 28 (Center-spot) [5]. A Type-2 placement for the 2,3-oxidation was generated at Rings eEc-eC1-eC4(eC3) plus Position 23 (Fig. 3F), followed by two Type-3 placements. These Type-3 placements had simultaneous contacts with the side-part to Bay-2 residue and with the bottom-flattened part to Bottom-residue, for the 7-oxidation (Fig. 3G) and 6-oxidation (Fig. 3H). The former, but not the latter, had Center-spot contact. The unidentified metabolite might correspond to the 7-hydroxy-AαC [44].

Precedent placements for the *N*-oxidation and 3-oxidation rather than the inhibition on rodent CYP1A1 Template, and also the primary sitting of the inhibitory molecule on human CYP1A1, would link to the strike differences in catalytic properties of human CYP1A1 and rodent CYP1A1 to AαC. Similar modes of interactions involving Pier-sitting and Sideway-route were expected on the basis of the distinct catalyses of human CYP1A1 and rodent CYP1A1 on MeAαC (Data not shown).

#### Differences on 2-amino-3-methylimidazo[4,5-*f*]quinoline (IQ)

Clear differences of human CYP1A1 and rat CYP1A1 are also known with the metabolic activation of IQ. Human CYP1A1 had 14-fold less activity of IQ *N*-oxidation than did rat CYP1A1 [45].

Only bi-molecule bindings, but not uni-molecule bindings, satisfied the simultaneous occupancy of Trigger-site and Site of oxidation for IQ placements on Template. Following descriptions are thus mostly on placements of the pro-metabolized molecules.

Several placements of IQ on CYP1A1 Template were generated for the *N*-oxidation, ring-oxidation and *N*-demethylation (Fig. 4). Plural placements using Pier-sitting were available selectively for rodent CYP1A1 at Rings eEc-eC2-eC1(C) (Fig. 4A), and at Rings eEc-eC2-eC4 plus near Position 22 (Fig. 4B) for both the initial 2,3-oxidation to yield the *N*^2^-hydroxylamine. An additional Pier-sitting was generated at Rings eEc-E-eC1(C) plus Positions 23 and 52’ for the *N*-demethylation on rodent CYP1A1 Template (Fig. 4C). Multiple Type-1 placements satisfying full occupancy of Ring eEc were generated on Template. A placement as the fourth preferred for rodent CYP1A1 and the primary for human CYP1A1 was available at Rings eEc-eC2-eC10 plus near Positions 7 and 22 (Fig. 4D). This placement was possible to mediate the *N*^*2*^-hydroxy metabolite formation through the initial 1,2-oxidation. The basic 1-nitrogen atom of imidazole, sitting at Site of oxidation, was however expected to interact with the oxidized form of heme-oxygen atom to work for the inhibition. The contribution for the *N*^*2*^-hydroxy metabolite formation was thus expected to be minimal, if any. A distinct Type-1 placement at Rings eEc-eC2-eC4(eC7)-eC6 (Fig. 4E) had the contact to Center-spot around Positions 27 and 28, and would contribute for both the 7-oxidation and inhibition through the interaction of the nitrogen atom with heme oxygen atom. An additional placement generated from Sideway-route of the migration was generated at Rings D-eEc-eC2(eC4)-eC10 for the 4,5-oxidation on rodent CYP1A1 Template (Fig. 4F). Ligands are able to migrate from Entrance-1 to a space around Rings D and E on rodent CYP1A1, but less abundantly on human CYP1A1 (Described below at the section of PAHs).

Couple placements occupying partly Ring eEc were also generated on both human and rodent CYP1A1Templates. Placements hanging at Position 33 were constructed at Rings eEc-eC2(eC10)-eC5-eC4(eC3) for the *N*-oxidation (Fig. 4G) and at Rings eEc-eC2(eC10)-eC1-eC4(eC3) for the *N*-demethylation (Fig. 4H). Both the placements were expected to be fastened with their contacts with Center-spot. These molecules were late in orders of placements of CYP1A1, but might contribute slightly the function of both human and rodent CYP1A1.

An additional placement was constructed at Rings eEc-E-eC1(eC3)-C (Fig. 4I) for the 7,8-oxidation. This placement had no rigid contact with Center-spot and also Trigger-residue descending would break the bi-molecule binding on rodent CYP1A1 Template.

These simulation results suggested the roles of Pier-sitting on the higher activation of IQ in rodent CYP1A1-system and of the preferred usage of the inhibitory placement in human CYP1A1-system. The placement using Pier-sitting described above would also contribute on the high abundance of the *N*-demethylation on rodent CYP1A1 Template, which was consistent with the in vivo data in mice [46].

IQ and 2-amino-3,4-dimethylimidazo[4,5-*f*]quinoline (MeIQ), but not 2-amino-3,8-dimethylimidazo[4,5-*f*]quinoxaline (MeIQx), undergo slightly CYP2C9-mediated activations to their mutagens [47].

A placement for the *N*^2^-oxidation was available at Rings J(M)-I(H)-D-C on CYP2C9 Template (Supplement Fig. 2A). The IQ molecule contacted simultaneously to Rear-wall with positions 8 and 9 of the quinoline part and the imidazole 1- and 2-amino parts, and also contacted to left-side borders at Positions 29 and 41 of CYP2C9 Template. Oxidation of the primary amino part was expected after the interaction with Trigger-residue at Ring D. Similar interaction was expected with MeIQ molecule on CYP2C9 Template (Supplement Fig. 2B). With the MeIQx molecule, Trigger-interaction would not be accomplished within the migration limit of Trigger-residue (Positions 7 and 28 at the junction of Rings C and D) on CYP2C9 Template, due to the Rear-wall contact with the 8-methyl part (Supplement Fig. 2C). Inability of Trigger-residue-mediated immobilization was likely to link to the lack of CYP2C9-mediated activation of MeIQx. A placement of IQ was constructed on CYP2C8 Template at Rings J(M)-I-(H)-D-C (Supplement Fig. 2D). No occupancy at Site of oxidation was observed after Trigger-residue-mediated left-side movement on the placement.

Contacts of the 2-amino-3-*N*-methyl-part to Rear-wall and of the position 7 part to Facial-wall generated a sitting for the *N*^2^-oxidation of IQ at Rings A(B)-D(C)-K on CYP3A4 Template (Supplement Fig. 2E), although the tight contact with Front-residue might be necessary to be fastened well after the trigger-interaction. The flipping of the 2-amino and 3-*N*-methyl parts generated a placement for the *N*-demethylation of IQ at Rings A(B)-D(E)-K (Supplement Fig. 2F). The *N*-demethylation of IQ was also expected from the placement of the pro-metabolized molecule at Rings E-eC1-eC2-eC4(eC5) on CYP1A2 Template (Supplement Fig. 2G).

Application of IQ molecule on CYP2E1 Template gave a placement at Rings C-A-eAB plus spaces above Ring eA and eAB (Data not shown). No occupancy at Site of oxidation was detected after Trigger-residue interactions of the uni-molecule placement. Another placement of IQ was constructed at Rings D-B(C)-B-eAB-eB after an anti-clock-wise rotation around 30°, which was the accessible limit of the 1-methyl part to Ring A. The rotation, however, resulted in the sitting of the *N*^6^- atom of IQ at Site of oxidation for the inhibition (Data not shown).”.

Although CYP1A1, CYP2C9 and CYP3A4 mediate the metabolic activation of IQ, CYP1A2 mediates mainly the activation of IQ to the *N*^2^-hydroxyl derivative in livers [48, 49]. Rat P-448 II-a (corresponding to CYP1A2) mediates 12-times efficiently the mutagenic activation of IQ than does rat P-448 II-d (corresponding to CYP1A1) [48].

Placements of IQ for the *N*^2^-oxidation were available at Rings eC1-eC4(eC3)-eC5 plus Position 5 of Ring E (Supplement Fig. 2H) and at Rings E-eC1(C)-eC2 plus Position 21 of Ring eE and Position 20 of Ring D (Supplement Fig. 2I). The former was selective on rodent CYP1A2 due to the N-Atom impedance at Position 31, and the latter was selective on human CYP1A2 due to the Ring eE sitting.

#### Differences on 2-amino-1,7-dimethylimidazo[4,5-*g*]quinoxaline (MeIgQx)

MeIgQx is activated by both human CYP1A1 and human CYP1A2 [50]. Oxidative metabolisms of MeIgQx were examined on both CYP1A1 and CYP1A2 Templates. Several placements of MeIgQx were generated on CYP1A1 Template. Plural placements using rodent CYP1A1-selective Pier-sitting were available at Rings eEc-eC1(eC2)-eC4 plus Positions 23 and 52’ (Fig. 5A) and at Rings eEc-eC1-eC3-eC6 plus Position 52’ and near Position 41 (Fig. 5B). In the presence of the trigger-molecule at Rings eB-B(A)-C-eC1-eC4, the former placement (Fig. 5A) suggested the *N*^2^-oxidation on rodent CYP1A1 Template. The latter suggested the 6-oxidation (thick stick-shape molecule, Fig. 5B), but the molecule entering from Entrance-2 was unable to pass a gate between Trigger and Bay-2 residues (cylindrical shape molecule, Fig. 5B).

A placement for the *N*-oxidation was available at Rings eEc(E)-eC2(eC1)-eC4(eC5)-eC6 plus Position 23 for the third rodent CYP1A1 and first human CYP1A1sittings (Fig. 5C). A Type-1 placement was constructed at Rings eEc-eC2-eC5(eC7)-eC8 plus Position 4 for the 6-oxidation and inhibition (Fig. 5D), but would have no significant functional contribution for the 6-oxidation due to the faint contact to Center-spot (Position 27–28) and inhibitory interaction with the oxidized form of heme. A distinct placement for linear tri-aromatics, like chlorpromazine and dibenzo-*p*-dioxin (oxanthrene) [5], was generated at Rings eEa(F)-eEc(E)-eC2(eC1) for the 4-oxidation on human CYP1A1 Template (Fig. 5E). A placement partly occupying Ring eEc for the *N*^2^-oxidation was generated at Rings eEc-eC2-eC4(eC5)-eC7 on CYP1A1 Template (Fig. 5F). An additional placement for the inhibition was constructed at Rings eEc-eC1-eC3 plus Position 23 and a Fjord space around Positions 40 and 10 (Data not shown), but was expected to interfere the descending of Trigger-residue.

MeIgQx was applied on both rodent CYP1A2 and human CYP1A2 Templates. Distinct uses of Ring eEa and Ring D, and also the sitting allowance of a basic nitrogen atom at Position 31 (N-atom impedance) cause the differences in regioselective metabolisms of human CYP1A2 and rodent CYP1A2 [6]. The *N*^2^-oxidations were expected from the pro-metabolized molecule-placements at Rings E-eC1eC4(eC3)-eC6 plus above Ring eC6 (Fig. 5G) and at Rings E-eC1(eC2)-eC4(eC3)-eC6 plus around Position 38’ (Fig. 5H) selectively on rodent CYP1A2 Template. The *N*^2^-hydroxy metabolite formation through the interaction at Position 21 of Ring eEa was available at Rings eEa-E-eC1(C)-eC3 plus around Positions 20 and 41 selectively on human CYP1A2 (Fig. 5I). Placements for the 6- and 7-methyl oxidations were constructed at Rings E-eC1-eC4-eC6 plus Position 20 and Entrance 2 (Data not shown) and at Rings E-eC1-eC4-eC5 plus Position 21 and Entrance 2 (Data not shown). Both placements were, however, expected to be non-functional due to the inhibitory interaction of the pyridine part at Position 5 on Template.

Placements of heterocyclic amines and differences of human CYP1A2 and rodent CYP1A2 on the metabolisms of Trp-P-1, Trp-P-2, MeAαC, MeIQx, and PhIP are discussed in our previous studies [6, 19].

#### Differences on PAHs

Human CYP1A1 and rat CYP1A1 oxidize benzo[a]anthracene at the 5,6-oxidation/8,9-oxidation ratio of 0.21 and 0.65, respectively [51]. Both enzymes metabolize benzo[c]phenanthrene at the 5,6- to 3,4-oxidation ratio of 1.48 and 4.26, respectively [51]. Differences are also observed on the benzo[a]pyrene (BaP) 4,5-oxidation [51, 52]. The 4,5-oxidation shares 0.8% of the total oxidation with human CYP1A1, and 8.5% with rat CYP1A1 [51]. In addition, the ratio of the 8,9-oxidation to the 11,12-oxidation of dibenzo[a,l]pyrene was less than 0.17 for human CYPA1 and 11 for rat CYP1A1 [21]. These experimental data on the quantitative differences in the regioselectivity imply the distinct preferred orders of placements on human and rodent CYP1A1 Templates.

Plural placements were generated for the 5,6-oxidation of benzo[a]anthracene at Rings D-E-eEc-eC2 (Sideway-route, Fig. 6A) and at Rings E-eEc-eC2-eC5 (Center-area, data not shown). The latter was, however, judged to be nonfunctional due to the lack of Center-spot interaction on CYP1A1 Template. A placement for the 8,9-oxidation of benzo[a]anthracene was available at Rings eEc-eC2-eC5-eC7 (Type 1, data not shown).

CYP1A1 mediates both 5,6-oxidation and 3,4-oxidation of benzo[c]phenanthrene [53]. Plural Placements of the pro-metabolized molecules were constructed for the 5,6-oxidation of benzo[c]phenanthrene, at Rings E-eEc-eC2-eC4 (Fig. 6B) and at Rings C-E-eEc-eC2 (Data not shown), The latter, however, had no Center-spot interaction. The 3,4-oxidation was expected from the pro-metabolized-molecule placement at Rings eEc-eC2-eC4-eC3 (Data not shown).

Plural placements for BaP 4,5-oxidation were generated at Rings D-E-eEc(eC1)-eC2 (Sideway-route, Fig. 6C) and at Rings E(eC1)-eEc(eC2)-eC5 (Data not shown). The latter placement was without Center-spot interaction in spite of the sitting at Center-area on Template.

Two distinct placements of the pro-metabolized molecules at Rings D-E(eC1-eC3)-eEc(eC2) (Fig. 6D) and at Rings E(eC1-eC3)-eEc-eC2-eC5 (Fig. 6E) were generated for the 8,9-oxidation of dibenzo[a,l]pyrene, together with the trigger molecule at A-B(C-eC3)-eD-D. Rat CYP1A1 showed the high regioselectivity on the 8,9-oxidation. Both the 7-oxidation and 11,12-oxidation of dibenzo[a,l]pyrene are rather selective with human CYP1A1. Rat CYP1A1 mediates the 11,12-oxidation, but only a lesser extent [21]. A placement for the 11,12-oxidation was generated around Center-area at Rings eEc-eC2(eC5-eC7)-eC4-eC3 (pro-metabolized molecule, Fig. 6F). A placement for the 6,7-oxidation was also constructed around Center-area at Rings eEc(eC2-eC4-eC6)-eC1-C (Data not shown), but the pro-metabolized-molecule sitting on Ring C would interfere the interaction of the trigger-molecule with the descending Fjord (Trigger)-molecule in similar to the cases with CYP1A2-mediated β-naphthoflavone (Fig. 1A and B) and 3-methylcholanthrene oxidations (Fig. 2 C and D). Instead, a Type-3 (bottom-flat) placement was generated for the 6,7-oxidation of dibenzo[a,l]pyrene at Rings eEc(E-eC1-eC3-eC4)-eC2-eC10 (Fig. 6G).

Among pro-metabolized molecule sittings of these PAHs, a mutual property became apparent on CYP1A1 Template. The differences in regioselectivities occurred on the sittings using Rings D and E on CYP1A1 Template, suggesting the preferred use of Rings D and E on rodent CYP1A1 Template.

The higher 9,10-oxidation of phenanthrene is also observed in rat and mouse CYP1A1 systems than in human CYP1A1 system [51, 54]. These results described above suggested consistently the more frequent uses of Rings D and E for PAH sittings on rodent CYP1A1 Template than on human CYP1A1 Template.

#### Differences on dibenzo-*p*-dioxin

On the placements of 2-chlorodibenzo-*p*-dioxin (2-chlorooxanthrene), an idea of the inhibitory molecule sitting at Rings eC2-eEc(E)-eEa-F (Supplement Fig. 3F) was proposed in our previous study on human CYP1A1 Template [5]. A distinct idea was proposed for the dominant rodent CYP1A1-mediated oxidation of 2-chlorodibenzo-*p*-dioxin as the use of Rings D and E sitting.

Rat CYP1A1 metabolized 2-chlorodibenzo-*p*-dioxin at 7-times higher rates than did human CYP1A1 [40]. A placement of 2-chlorodibenzo-*p*-dioxin was available for the 1,2-oxidation using Rings D-E-eEc, in which the 2-chloro part sat at Position 52’ (Supplement Fig. 3G). Another placement using Position 52’ was also generated at Rings eEc-eC1-eC3 plus Position 52’ (Supplement Fig. 3H). The preferred use of Rings D and E and/or Pier-sitting rather to the Type-2 or Type-3 placements [5] was expected to contribute on the distinct catalytic properties of rodent CYP1A1 and human CYP1A1for chlorinated dibenzo-*p*-dioxins [40].

### Enhancement and co-mutagenicity

#### 7,8-Benzoflavone and BaP interaction on CYP3A4

Activation of monooxygenases by 7,8-benzoflavove (α-naphthoflavone) was studied historically as indirect evidence suggesting multiple monooxygenases catalyzing the oxidations of chemicals in livers of human [55] and experimental animals [56]. This phenomenon was then studied as a model to understand the cooperativity and multiple substrate-binding sites using recombinant CYP enzymes particularly CYP3A4 [57, 58]. A mechanistic basis of the activation was proposed from results of CYP3A4 Template system established [11]. The general scheme for interactions of ligands with CYP3A4 Template is described in the section of CYP3A4 of Materials and Methods. CYP3A4 ligands need to fulfill three essential contacts, at Site of oxidation (Position 6–7), Trigger-site (Position 26) and at least a part of Rings I, J, K for IJK-Interaction on CYP3A4 Template. CYP3A4 ligands interact with Template in ways of uni-molecule and bi-molecule bindings. In cases of bi-molecule binding, ligands sitting at Site of oxidation are termed pro-metabolized molecules. The molecules are necessary to occupy both Site of oxidation and at least a part of Rings I-J-K region for I-J-K-Interaction to receive the facial-side pushing. Another molecule termed trigger molecule occupies Trigger-site (Position 26). Both pro-metabolized and trigger molecules are required to stay simultaneously within Width-gauge, and have a slight over-lapping point(s) on Template. Cavity-2 (Trigger) residue would descend after ligand-occupancy at Trigger-site (Position 26) and then immobilize ligands to trigger the catalyses in the Template system. Two molecules of identical ligands behave as pro-metabolized and trigger molecules in most of bi-molecule bindings.

Aryl hydrocarbon hydroxylation (AHH) is an indicator of CYP1A induction and composed mainly of 3-hydroxy-BaP production. Placements for CYP3A4-mediated 2,3-oxidation of BaP were constructed as uni-molecule bindings on Template. A placement at Rings A-B(D)-C-L (Fig. 7A) had poor I-J-K-Interaction. Another placement at Rings A-E(D)-K-Q (Fig. 7B shown as cylindrical shape) fulfilled three essential contacts as a uni-molecule binding but not rigid in the trigger interaction at Position 26. On the same pro-metabolized-molecule placement of BaP, 7,8-benzoflavone molecule was expected to act also as the trigger molecule at Rings D-C-L-P-Y plus above Bay-2 residue (Fig. 7C). Couple placements including at Rings C-L-P-X-W plus Position 62 and at Rings C-D-K-Q-W plus Position 30 (data not shown) were also constructed for the trigger molecule but these of 7,8-benzoflavone molecules were less likely to take parts due to the sitting instability at Trigger-residue descending, steric repulsions with the pro-metabolized molecule, and difficulty to pass through Cavity-2 (Trigger) residue. The bi-molecule binding with 7,8-benzoflavone at Rings D-C-L-P-Y plus above Bay-2 residue would offer the rigid immobilization of BaP pro-metabolized molecule after the trigger-action of Cavity-2 (Trigger) residue (Fig. 7C), and thus support the efficient catalysis observed as 7,8-benzoflavone-supported activation of BaP oxidation (AHH).

#### 7,8-Benzoflavone and benzo[e]pyrene 9,10-dihydrodiol on CYP3A4

Metabolic activations of benzo[e]pyrene (BeP) 9,10-dihydrodiol to mutagens toward Salmonella typhimurium strain TA 100 is reported in the presence of hepatic microsomes from humans [59]. In the absence of 7,8-benzoflavone, hepatic microsomes only weakly activated BeP 9,10-dihydrodiol to mutagens. 7,8-Benzoflavone enhanced an 8-fold the human-liver-mediated metabolic activation. A compound unable to yield a bay-region diol epoxide, 9,10-dihydroxy-9,10,11,12-tetrahydro-BeP is not metabolically activated to mutagenic metabolites in the presence or absence of 7,8-benzoflavone [59].

A placement of BeP 9,10-dihydrodiol on CYP3A4 Template was available at rings A-E(K)-F(J) for the pro-metabolized molecule. 7,8-Benzoflavone sat at Rings D-C-L-Q-W plus Position 30 for the trigger-molecule (Fig. 7D). The position 11 of this dihydrodiol sat at Site of oxidation of CYP3A4 and would be oxidized to yield the 9,10-dihydrodiol-11,12-oxide. A placement for the 2,3-oxidation was generated at Rings A-D(K)-E-F (Fig. 7E) as the uni-molecule and bi-molecule binding with 7,8-benzoflavone molecule. The 2,3-oxide production might thus contribute on the mutagenic activation slightly.

The sitting of this dihydrodiol on CYP1A1 Template was generated as bi-molecule binding at Rings eEc-eC2(eC4)-eC10(eC5) for the pro-metabolized molecule and at Rings A(eB-eD)-B-C-eC3 for the trigger molecule (Data not shown), but the both molecules contacted at single site at Position 29 on the Template. The replacement of the trigger molecule with 7,8-benzoflavone at Rings A-B-C(D)-eC1-eC4 increased the overlapping at Rings eC1 and eC4 on Template (Fig. 7F). Both CYP3A4 and CYP1A1 were possible to contribute on the dihydrodiol activation, but CYP3A4 would mediate mainly this activation due to the clear difference in the expression levels in human livers.

#### Norharman and dibenzo[a,e]fluoranthene on CYP1As

Norharman is also known as an effector of metabolic activations of various chemicals [60]. This chemical altered microsomal oxidations of dibenzo[a,e]fluoranthene (DBF). The formations of 3,4-dihydrodiol-DBF, 12,13-dihydrodiol-DBF and 7-hydroxy-DBF were more than 8-, 3- and 4-times higher in the presence of 0.6 mM norharman in liver microsomes of 3-methylcholanthrene-treated rats, respectively [61].

To verify the role of CYP1A enzymes, DBF molecule was applied at first on CYP1A2 Template. Both the molecules for the 3,4-oxidation at Rings eE-E(eC1-eC4)-C-B) plus above Position 10, and for the 12,13-oxidation at Rings E-eC1(eC3)-eC4-eC5 plus left-side of Ring eC3 did not stay within CYP1A2 Template area. Parts of both molecules exceeded to Fjord area (Data not shown). A placement for the 6,7-oxidation was constructed at Rings eE-E-C(D-B)-eC1-eC2-eC4 (Data not shown). This molecule did not pass the gate between Bay-1 and Fjord (Trigger) residues from Entrance 1, and also had negligible chances to migrate from Entrance 2. These simulation results suggested the poor catalyses of CYP1A2 on DBF oxidations.

Dibenzo[a,e]fluoranthene molecule sat on CYP1A1 Template for the 3,4–12,13- and 6,7-oxidations at Rings eEc-eC2(eC4-eC6)-eC5-eC9(eC8) (Fig. 7G, cylindrical shape), at Rings eEc-eC2(eC4-eC3-eC6)-eC5-eC9 (Fig. 7H, cylindrical shape), and at Rings eEc-eC2(eC4-eC3-eC6)-eC5(eC10)-eC9 (Data not shown) as pro-metabolized molecules, respectively. The placement for the 6,7-oxidation exceeded the border of Template area at Ring eC9 and would thus be adapted after the hitting to right-side wall. A uni-molecule placement for the 2,3-oxidation at Rings eEc-eC1(C-B)-eC3-eC6 plus left-side of Ring eC6 (Fig. 7I) fulfilled three essential contacts but lacked the hanging at Position 33 of Ring eC2. In addition, the migration from Entrance 2 to Trigger-site (Position 10–11) was less likely to occur. Three bi-molecule placements required trigger molecules for the functional contributions for the 3,4-, 6,7- and 12,13-oxidations. A possible trigger-molecule was constructed at Rings F-D(eD-eB)-B-C-eC3(eC4)-eC6 (Fig. 7G, stick shape), if the sitting at Ring F was allowed. The trigger molecule of dibenzo[a,e]fluoranthene was unable to pass the gate between Bay-1 and Trigger residues (Gatekeeper) due to the bulky size [19]. The chance to migrate from Entrance 2 was also expected to be very low for this trigger molecule. Norharman molecule at rings B-C-eC1(eC3) (Fig. 7H, stick shape) was able to substitute dibenzo[a,e]fluoranthene molecule for trigger molecule. Norharman molecule would enter without the difficulty from Entrance 1 to serve for the trigger-action.

Dibenzo[a,e]fluoranthene is mutagenic at the thymidine kinase locus assay on h1A1v2 cells, which are human B-lymphoblastoid cells transfected with human CYP1A1 cDNA [62].

#### Norharman and Trp-P-2 on CYP1A2

In Salmonella mutagenesis test of Trp-P-1 and Trp-P-2, the numbers of revertant induced are increased in the presence of norharman [60]. Higher capacities of CYP1A2 rather than CYP1A1 on Trp-P-2 activation were observed in their preparations of rats [63] and humans [64].

Plural placements for the *N*^2^-oxidation of Trp-P-2 were generated as the bi-molecule binding on CYP1A2 Template. A placement for the pro-metabolized molecule was available at Rings eC1-eC4-eC6 plus Position 5 of Ring E, Position 9 of Ring C and a space around Position 39’, and at Rings A(eB)-B-C plus Position 47’ as a trigger molecule on both human and rodent CYP1A2 (Fig. 8A). Another placement was constructed at Rings eC1-eC3-eC6 plus Position 5 of Ring E, Position 25 of Ring eC4 as a pro-metabolized molecule (Fig. 8B). The occupancy of a basic nitrogen atom at Position 31 was allowed on rodent CYP1A2 Template, but not on the human CYP1A2 (N-atom impedance) [6]. In these bi-molecule bindings, 2-amino part of the trigger molecule at Position 47’ was at a close contact with Bay-1 residue. The steric difficulties on trigger-action would cause the inefficient catalyses particularly on rodent CYP1A2 Template, due to the difference in accommodation space at Bay-1 between human CYP1A2 and rodent CYP1A2 [6]. Norharman molecule at Rings A(eB)-B-C had no steric difficulty to serve for the trigger-action (Fig. 8C).

#### Norharman and aniline on CYPs

Activation of norharman on aniline analogues are known as a comutagenic action [60, 65]. Formation of a fused product 9-(4’-amino-3’-methylphenyl)-9H-pyrido[3,4-*b*]indole (aminophenylnorharman), is characterized during the metabolism of aniline with a comutagen, norharman [66, 67]. Several CYP enzymes including CYP1A1, CYP1A2 and CYP3A4, but not CYP2C9 and CYP2C19, supported the aminophenylnorharman formation [67].

A placement of aniline molecule at Rings eEc plus Position 33 (Type-1 sitting) with norharman at B-C-eC1(E) was available on CYP1A1 Template (Fig. 8D). Aniline molecules at Rings E plus at Position 21 and at Ring E plus Position 31 were generated on human CYP1A2 and rodent CYP1A2 Templates, respectively (Fig. 8E). Norharman molecule served as trigger-molecule at Rings B-C-eC1 for these CYP1A2 interactions. Aniline molecule at Rings A plus 6’ (Groove) and norharman molecule at Rings C-D-E(K) interacted on CYP3A4 Template (Fig. 8F). These placements would offer the chances to yield aminophenylnorharman through *C*-oxidations of aniline molecule. On CYP2C9 and CYP2C19 Templates, pro-metabolized molecules were necessary to contact to Rear-wall simultaneously with the plural positions. Aniline molecules were unable to fulfill both the requirements of sitting at Site of oxidation and simultaneous plural-contact to Rear wall on CYP2C9 and CYP2C19 Templates (Data not shown). The lack of the available aniline-placements on CYP2C9 and CYP2C19 Templates was consistent with the experimental data [67].

The *N*-oxidation of aminophenylnorharmans is necessary for the second step of the mutagenic activation. CYP1A1, CYP1A2 and CYP2C9 were suggested to mediate the activation using UMU test [68].

A placement of aminomethylphenylnorharman for the *N*-oxidation was generated at Rings eEc-eC2-eC4-eC7(eC6)-eC8 plus Position 53 as the pro-metabolized molecule and at Ring A-B(C-eC3)-D(eD) plus Positions 27 and 30 as the trigger-molecule on CYP1A1 Template (Fig. 8G). A similar bi-molecule placement was constructed for the *N*-oxidation of aminophenylnorharman on CYP1A1 Template (Data not shown). The CYP1A1-mediated mutagenic activation of aminophenylnorharman was, however, not detected in the UMU test [68]. The exact reason to yield the differences between both aminophenylnorharmans was unclear, but the rotation of the non-substituted anilino part at Ring eC2 might cancel the hanging at Position 33 to lead the loss of the functional contribution (Data not shown).

A placement for the *N*-oxidation was available on CYP1A2 Template at Rings E-eC1-eC4-eC6 plus both side spaces of Ring eC6 as the pro-metabolized molecule and at Rings C-B(A-eA)-eB(eD) plus a space right side of Ring eA as the trigger-molecule (Fig. 8H). The rotation of the anilino part was restricted with the trigger molecule.

CYP2C9 does not mediate the formation of aminophenylnorharman, but supports the mutagenic activation. A placement for the *N*-oxidation was available at Rings J-I(K)-D(E)-C (Fig. 8I). Trigger-residue would support the contact to the left-side border at Rings I and J, and also fasten the anilino part at Ring J.

## Discussion

From simulation experiments of β-naphthoflavone and 3-methylcholanthrene on CYP-Template systems, both β-naphthoflavone and 3-methylcholanthrene were found to rely heavily on non-constitutive CYP1A1 enzyme for their oxidative metabolisms. Most of PAHs, such as BaP and benzo[a]anthracene, are metabolized by both CYP1A1 and constitutive CYP1A2, in liver of experimental animals and humans. Higher dependence of β-naphthoflavone and 3-methylcholanthrene on CYP1A1-mediated oxidations would favor their relatively extended-stay in hepatocytes until the accumulation of newly synthesized CYP1A1 protein. These properties of β-naphthoflavone and 3-methylcholanthrene would contribute to the efficient biosynthesis and accumulation of CYP1A1 and CYP1A2 in livers.

Both β-naphthoflavone and 3-methylcholanthrene fulfilled the three-essential contacts on CYP1A2 Template as bi-molecule bindings. The indecisive bindings of the pro-metabolized molecules of both β-naphthoflavone and 3-methylcholanthrene at Trigger-site (Position 10) would, however, cause the collapse of the bi-molecule bindings at the descending of Trigger-residue on CYP1A2 Template (Fig. 1A and B, and 2 C and D). These modes of interaction would cause the substantial lack of CYP1A2 contribution on the oxidative metabolisms of β-naphthoflavone and 3-methylcholanthrene.

No excretion of β-naphthoflavone itself was detected in the urine after the i. v. administration of 10 mg/kg of β-naphthoflavone or the infusion for 2 h at the rate of 6 mg/hr/kg [69], suggesting the role of hepato-biliary route for the clearance through the initial oxidative metabolism. High blood clearance (less than 40 min of the half-life) and large volume of distribution (6 L/kg b. w.) are observed in rats [69], suggesting the liver and peripheral tissue deposit, and slow release of β-naphthoflavone in untreated rats.

In addition to these in vivo pharmacokinetic profiles, the present study suggests CYP1A1-selectivity of both β-naphthoflavone and 3-methylcholanthrene on their metabolisms as a key factor for excellent inducers.

A clear difference has been known on the inhibition of CYP1A2 between β-naphthoflavone and a related α-naphthoflavone (7,8-benzoflavone). Although the inhibition constant of β-naphthoflavone on human CYP1A2 remains unclear, substantial lack of the CYP1A2-interaction (Fig. 1) may suggest the trivial inhibitory property.

Two placements of α-naphthoflavone were available on CYP1A2 Template. A placement of the 5,6-oxidation was generated at Rings D-E-eC1(eC2)-eC3-eC6 for the pro-metabolized molecule and at Rings C-B-A(eB)-eA plus a space above of Ring eA for trigger molecule (Data not shown). The sandwich-type interaction with Bay-2 residue might retard the product release. Another placement was constructed at Rings E-D-B(eB)-A plus a space right-side above Ring A (Data not shown). The rotatable phenyl part sitting above Ring A would interfere the descending/ascending of Fjord(Trigger)-residue. The latter placement was expected to contribute mainly the inhibition of α-naphthoflavone on CYP1A2.

Extrapolation of toxicological results in rodents including the carcinogenicity and mutagenicity to humans are a critical step for the safety evaluations. Oxidative and reductive metabolisms of chemicals affect their exposures in the body and alter the appearance of their toxicological events. Several enzymes of CYP family mediate these biological processes in liver and extra-hepatic tissues. Assessments of the metabolisms at individual CYP enzyme levels are thus necessary to evaluate rigidly the safety of chemicals.

Clear differences are known on the oxidative metabolisms of PAHs, arylamines and dioxins between rodent CYP1A1 and human CYP1A1. In the present study, these differences are explained as the altered preference of two-types of placement usages. One is Pier-sitting (Position 52’), which is selective on rodent CYP1A1 Template (Figs. 3, 4 and 5). Another is the use of Ring-D/E sitting, which is also predominant on rodent CYP1A1 Template (Fig. 6). Instead, preferred uses of Rings eEc-eEa-F are observed on Templates of human CYP1A1 than rodent CYP1A1 with dioxins and chlorpromazine [5]. These guides for distinct orders of placements are useful to assume relative abundances of regio-isomeric metabolites as well as the distinct catalyses of human and rodent CYP1A1 enzymes. Clear differences in regio-selectivity are also known between human and rodent CYP1A2 enzymes. The differences are also explained as distinct uses of placements on both human and rodent CYP1A2 Templates [6].

Additions of hydrophobic chemicals often result in the inhibitions of CYP-mediated metabolisms of substrates, but sometimes rather enhance metabolisms of the substrates even in vivo [70]. This enhanced metabolism had been investigated as heterotropic cooperativity [58, 71, 72] or allosteric interaction [73]. We first proposed a simultaneous bi-molecule binding of small-sized ligands in a way of pro-metabolized and trigger molecules as normal phenomena of CYP1A2, from the results of simulation experiments to explain regio-selective oxidations on Template [19]. Many catalytic reactions are now explained as sittings of two identical-molecules as pro-metabolized- and trigger-molecule combination on CYP Templates. Enhancement phenomena described above were explained as bi-molecule placements of distinct ligands as pro-metabolized and trigger molecules. 7,8-Benzoflavone(α-naphthoflavone) on CYP3A4 Template (Fig. 7C and D), and norharman on both CYP1A2 (Fig. 8 C and E) and CYP1A1 Templates (Figs. 7H and 8D) serve as typical trigger molecules for the enhancements. A mutual property existed on these activation events as compensations for poor or inefficient actions of the trigger-molecule in bindings of two identical molecules. These events included supplement of trigger-molecule (Fig. 8D-F), rigid interaction with Trigger-residue (Cavity-2 residue) (Fig. 7C), Gate passing (Fig. 7H) and Bay-1 interaction (Fig. 8A-C). Similar phenomena to escape obstacles or hinderances on the bi-molecule bindings of ligands were also observed on the activation of CYP2C9 [8], CYP2E1 [10] and CYP3A4 [11]. Relatively low affinities of 7,8-benzoflavone for CYP3A4 [74] and of norharman for CYP1A2 [75] as pro-metabolized molecules may also contribute for the modulating properties.

### Electronic supplementary material

Below is the link to the electronic supplementary material.

Supplement Fig. 1 Typical hexagonal-fused grid Templates of CYP enzymes. Template systems of CYP1A1 (A and B), CYP1A2 (C and D), CYP2E1 (E), CYP3A4 (F), CYP2C9 (G), and CYP2C19 (H) are shown with their Ring and Position numbers. Allowable width of ligands is indicated as Width-gauge. Ligands are shown as 3D-structures as indicated in B and D. Thick grey arrows at the bottom of Width-gauge indicate the access points of heme-oxygen

Supplement Fig. 2 Interactions of IQ, MeIQ and MeIQx on CYP2C9, CYP2C8, CYP3A4 and CYP1A2 Templates. Placements on Templates of CYP2C9 for IQ, MeIQ and MeIQx *N*-oxidations (A-C), of CYP2C8 for IQ *N*-oxidation (D), of CYP3A4 for IQ *N*-oxidation (E) and *N*-demethylation (F), and of CYP1A2 for the IQ *N*-demethylation (G) and *N*-oxidations (H and I). Dark arrows indicate the possible causes of defects

Supplement Fig. 3 Rodent CYP1A1-selective Pier-sitting (Position 52’). Placements of PCB126 for the 4-OH-TCB (A and B) and 4-OH-PCB formations (C), of naringenin for the inhibition (D and E), and of 2-chlorodibenzo-*p*-dioxin (F, G and H) on CYP1A1 Template

## Data Availability

All generated data are included in this manuscript, or were obtained from peer-reviewed articles cited in the literature.

## Abbreviations

AαC: 2-amino-9H-pyrido[2,3-*b*]indole
aminophenylnorharman: 9-(4’-aminophenyl)-9H-pyrido[3,4*-b*]indole or 4-(9H-β-carbolin-9-yl)aniline
BaP: Benzo[a]pyrene
BeP: Benzo[e]pyrene
CYP: Cytochrome P450
DBF: Dibenzo[a,e]fluoranthene
GDC-0339: 5-amino-N-{5-[(4R,5R)-4-amino-5-fluoro-1-azepanyl]-1-methyl-1H-pyrazol-4-yl}-2-(2,6-difluorophenyl)-1,3-thiazole-4-carboxamide
IQ: 2-amino-3-methylimidazo[4,5-*f*]quinoline
MeAαC: 2-amino-3-methyl-9H-pyrido[2,3*-b*]indole
MeIgQx: 2-amino-1,7-dimethylimidazo[4,5-*g*]quinoxaline
MeIQx: 2-amino-3,8-dimethyl-3H-imidazo[4,5-*f*]quinoxaline
PAH: polyaromatic hydrocarbon
PCB126: 3,3′,4,4′,5-pentachlorobiphenyl
PhIP: 2-amino-1-methyl-6-phenylimidazo[4,5-*b*]pyridine
Trp-P-1: 3-amino-1,4-dimethyl-5H-pyrido[4,3-*b*]indole
and Trp-P-2: 3-amino-1-methyl-5H-pyrido[4,3-*b*]indole

## Footnotes

1 Capital letters are used for Template-associated terms to distinguish from descriptions of chemical- and enzyme-related events.
