# Supplementary material for: Application of fused-grid-based CYP-Template systems for genotoxic substances to understand the metabolisms
Source: Genes Environ. 2023 Aug 7;45:22. doi: 10.1186/s41021-023-00275-4 (PMC10405451; doi:10.1186/s41021-023-00275-4)
Supplement: Supplementary file 1 — Supplement Fig. 1 Typical hexagonal-fused grid Templates of CYP enzymes. Template systems of CYP1A1 (A and B), CYP1A2 (C and D), CYP2E1 (E), CYP3A4 (F), CYP2C9 (G), and CYP2C19 (H) are shown with their Ring and Position numbers. Allowable width of ligands is indicated as Width-gauge. Ligands are shown as 3D-structures as indicated in B and D. Thick grey arrows at the bottom of Width-gauge indicate the access points of heme-oxygen [file 41021_2023_275_MOESM1_ESM.pdf]

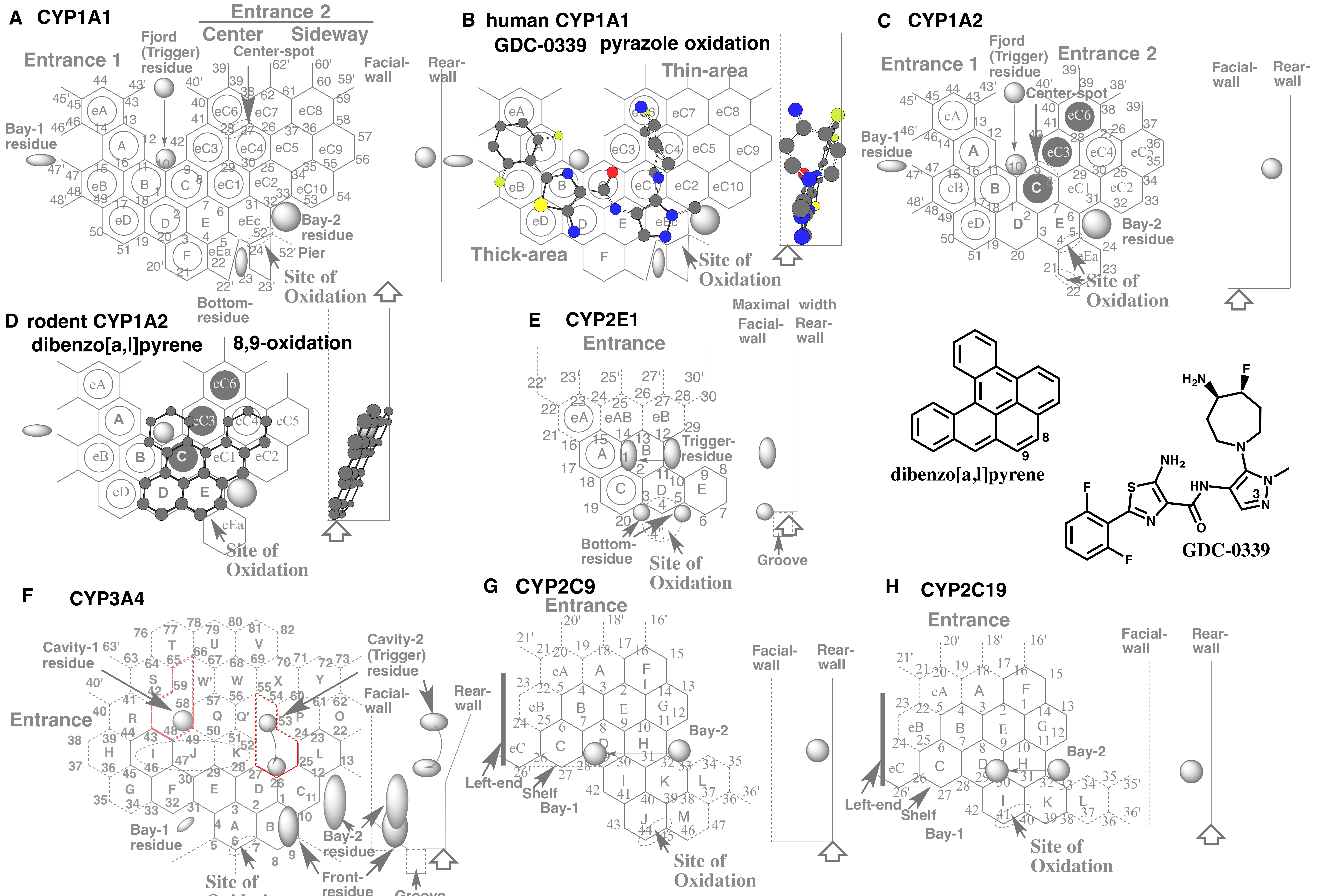
A CYP1A1
Entrance 2
Center
Sideway
Center-spot
Fjord (Trigger) residue
Entrance 1
Facial-wall
Rear-wall
Bay-1 residue
Bay-2 residue
Pier
Site of Oxidation
Bottom-residue
B human CYP1A1
GDC-0339 pyrazole oxidation
Thin-area
Thick-area
Site of Oxidation
C CYP1A2
Entrance 1
Fjord (Trigger) residue
Entrance 2
Center-spot
Facial-wall
Rear-wall
Bay-1 residue
Bay-2 residue
Site of Oxidation
D rodent CYP1A2
dibenzo[a,l]pyrene
8,9-oxidation
Site of Oxidation
E CYP2E1
Entrance
Maximal width
Facial-wall
Rear-wall
Trigger-residue
Bottom-residue
Site of Oxidation
Groove
dibenzo[a,l]pyrene
GDC-0339
F CYP3A4
Cavity-1 residue
Cavity-2 (Trigger) residue
Entrance
Facial-wall
Rear-wall
Bay-1 residue
Bay-2 residue
Site of Oxidation
Front-residue
Groove
G CYP2C9
Entrance
Facial-wall
Rear-wall
Bay-2
Left-end
Shelf
Bay-1
Site of Oxidation
H CYP2C19
Entrance
Facial-wall
Rear-wall
Bay-2
Left-end
Shelf
Bay-1
Site of Oxidation

SupFig 1
