# Supplementary figures and images for: Application of fused-grid-based CYP-Template systems for genotoxic substances to understand the metabolisms
Source: Genes Environ. 2023 Aug 7;45:22. doi: 10.1186/s41021-023-00275-4 (PMC10405451; doi:10.1186/s41021-023-00275-4)

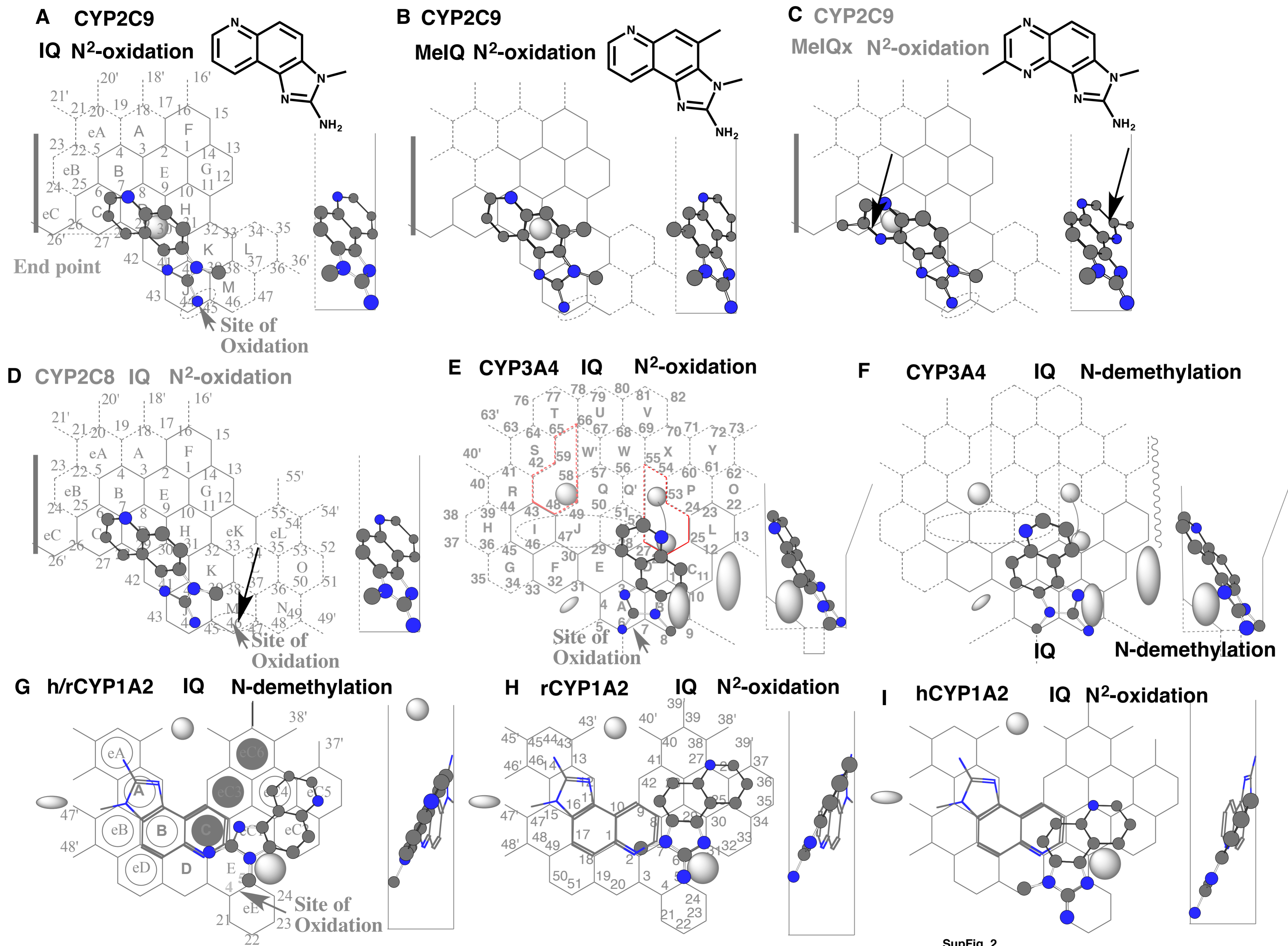

SupFig. 2

Supplement: Supplementary file 2 — Supplement Fig. 2 Interactions of IQ, MeIQ and MeIQx on CYP2C9, CYP2C8, CYP3A4 and CYP1A2 Templates. Placements on Templates of CYP2C9 for IQ, MeIQ and MeIQx N-oxidations (A-C), of CYP2C8 for IQ N-oxidation (D), of CYP3A4 for IQ N-oxidation (E) and N-demethylation (F), and of CYP1A2 for the IQ N-demethylation (G) and N-oxidations (H and I). Dark arrows indicate the possible causes of defects [file 41021_2023_275_MOESM2_ESM.pdf]
