# Supplementary material for: Application of fused-grid-based CYP-Template systems for genotoxic substances to understand the metabolisms
Source: Genes Environ. 2023 Aug 7;45:22. doi: 10.1186/s41021-023-00275-4 (PMC10405451; doi:10.1186/s41021-023-00275-4)
Supplement: Supplementary file 3 — Supplement Fig. 3 Rodent CYP1A1-selective Pier-sitting (Position 52’). Placements of PCB126 for the 4-OH-TCB (A and B) and 4-OH-PCB formations (C), of naringenin for the inhibition (D and E), and of 2-chlorodibenzo-p-dioxin (F, G and H) on CYP1A1 Template [file 41021_2023_275_MOESM3_ESM.pdf]

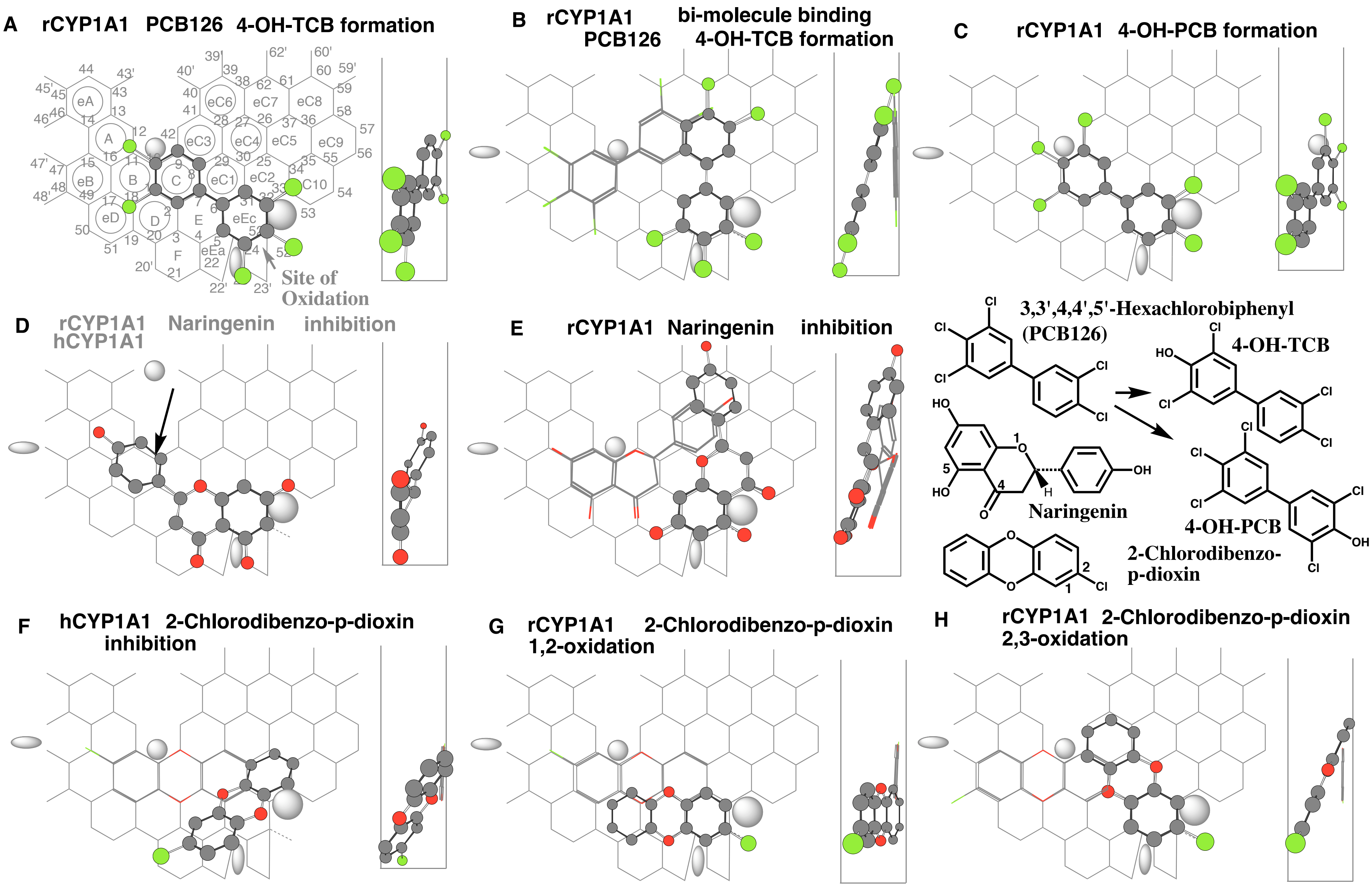

Supplement Fig. 3 Rodent CYP1A1-selective Pier-sitting (Position 52')

Placements of PCB126 for the 4-OH-TCB (A and B) and 4-OH-PCB formations (C), of naringenin for the inhibition (D and E), and of 2-chlorodibenzo-p-dioxin (F, G and H) on CYP1A1 Template
